# Genome-wide association study in Japanese females identifies fifteen novel skin-related trait associations

**DOI:** 10.1038/s41598-018-27145-2

**Published:** 2018-06-12

**Authors:** Chihiro Endo, Todd A. Johnson, Ryoko Morino, Kazuyuki Nakazono, Shigeo Kamitsuji, Masanori Akita, Maiko Kawajiri, Tatsuya Yamasaki, Azusa Kami, Yuria Hoshi, Asami Tada, Kenichi Ishikawa, Maaya Hine, Miki Kobayashi, Nami Kurume, Yuichiro Tsunemi, Naoyuki Kamatani, Makoto Kawashima

**Affiliations:** 1 Department of Dermatology, School of Medicine, Tokyo Women’s Medical University, Shinjuku, Tokyo, 162-8666 Japan; 2 StaGen Co., Ltd., Taito-ku, Tokyo, 111-0051 Japan; 3 EverGene Ltd., Shinjuku-ku, Tokyo, 163-1435 Japan; 4 Life Science Group, Healthcare Division, Department of Healthcare Business, MTI Ltd., Shinjuku-ku, Tokyo, 163-1435 Japan; 5 LunaLuna Division, Department of Healthcare Business, MTI Ltd., Shinjuku-ku, Tokyo, 163-1435 Japan

## Abstract

Skin trait variation impacts quality-of-life, especially for females from the viewpoint of beauty. To investigate genetic variation related to these traits, we conducted a GWAS of various skin phenotypes in 11,311 Japanese women and identified associations for age-spots, freckles, double eyelids, straight/curly hair, eyebrow thickness, hairiness, and sweating. *In silico* annotation with RoadMap Epigenomics epigenetic state maps and colocalization analysis of GWAS and GTEx Project eQTL signals provided information about tissue specificity, candidate causal variants, and functional target genes. Novel signals for skin-spot traits neighboured *AKAP1*/*MSI*2 (rs17833789; *P* = 2.2 × 10^−9^)*, BNC*2 (rs10810635; *P* = 2.1 × 10^−22^)*, HSPA1*2*A* (rs12259842; *P* = 7.1 × 10^−11^), *PPARGC1B* (rs251468; *P* = 1.3 × 10^−21^), and *RAB11FIP*2 (rs10444039; *P* = 5.6 × 10^−21^). *HSPA1*2*A* SNPs were the only protein-coding gene eQTLs identified across skin-spot loci. Double edged eyelid analysis identified that a signal around *EMX*2 (rs12570134; *P* = 8.2 × 10^−15^) was also associated with expression of *EMX*2 and the antisense-RNA gene *EMX*2*OS* in brain putamen basal ganglia tissue. A known hair morphology signal in *EDAR* was associated with both eyebrow thickness (rs3827760; *P* = 1.7 × 10^−9^) and straight/curly hair (rs260643; *P* = 1.6 × 10^−103^). Excessive hairiness signals’ top SNPs were also eQTLs for *TBX15* (rs984225; *P* = 1.6 × 10^−8^), *BCL*2 (rs7226979; *P* = 7.3 × 10^−11^), and *GCC*2 and *LIMS1* (rs6542772; *P* = 2.2 × 10^−9^). For excessive sweating, top variants in two signals in chr2:28.82-29.05 Mb (rs56089836; *P* = 1.7 × 10^−11^) were eQTLs for either *PPP1CB* or *PLB1*, while a top chr16:48.26–48.45 Mb locus SNP was a known *ABCC11* missense variant (rs6500380; *P* = 6.8 × 10^−10^). In total, we identified twelve loci containing sixteen association signals, of which fifteen were novel. These findings will help dermatologic researchers better understand the genetic underpinnings of skin-related phenotypic variation in human populations.

## Introduction

Skin phenotypes such as freckles, hairiness, and excessive sweating can be serious problems for some individuals. Investigations among Japanese women (*n*~854 to 4345) for particular beauty problems revealed the following concerns broken down by individuals in particular age ranges and in order of significance: 20s) dry skin, large pores, and acne; 30s) freckles, dry skin, and large pores; 40s) freckles, wrinkles, and sagging skin; and 50s and older) wrinkles, freckles, and sagging skin^1,2^. These concerns often impact an individual’s Quality-of-Life (QOL), and there have been major developments in medical cosmetic treatment and the cosmetic industry in recent years to address these problems.

Review of patient family histories has suggested that genetic factors may be related to some of these phenotypes, and a number of studies have revealed potential causative genes for certain skin-related phenotypes. The thickness of human hair fibers is very differentiated across world-wide populations, and almost a decade ago, researchers identified its association with a non-synonymous variant in the ectodysplasin A receptor gene (*EDAR*)^3,4^. Recently, it was shown that the A481T and H615R alleles of *OCA*2, which is a causal gene for oculocutaneous albinism, are correlated with the skin color of Japanese people^5–7^. Several genome-wide association studies (GWAS) and candidate gene analyses in European ancestry population samples have identified variants associated with skin pigmentation in or near *ASIP*, *HERC*2, *IRF4*, *MC1R*, *OCA*2, *SLC*2*4A4*, *TYR*, and *BNC*2^8–10^, while *IRF4*, *MC1R*, *ASIP*, and *BNC*2 were also found to be associated with freckles and facial pigmented spots^11,12^. Associations were also identified for *MC1R* and *ASIP* with red hair color, *TCHH* and *WNT10A* with hair curl, and *OCA*2, *IRF4*, *SLC45A*2, *SLC*2*4A4*, and *MC1R* with blond versus brown hair color. A more recent 23andMe GWAS report of 42 human phenotypes found multiple loci associated with skin-related traits such as male-pattern baldness, unibrow, chin dimples, and nose size^13^. However, most large-scale association studies have been performed in population samples with predominantly European ancestry. Analysis of traits in multiple ethnicities is an important part of modern GWAS analyses^14–16^, and a GWAS analysis of multiple skin-related traits in an East Asian population sample should help identify new loci and confirm and or refine previously reported association signals from those studies. If new genes and variants related to these phenotypes can be identified, it may be possible to decrease cosmetic problems and medical care costs by providing instructions on lifestyle habits, diet, and makeup, as well as preventive measures such as use of custom-made cosmetics and therapeutic intervention in the initial stage of symptom development.

Here, we report on a GWAS of skin-related phenotypes that analyzed over eleven-thousand Japanese females and identified both novel loci and refined known association signals. Use of epigenetic state data combined with colocalization analysis of GWAS and eQTL signals allowed us to narrow down the lists of candidate causal variants, identify likely target genes, and better understand the biologic functions of a number of genes with important effects that were detected in this study.

## Results

**Figure 1:**
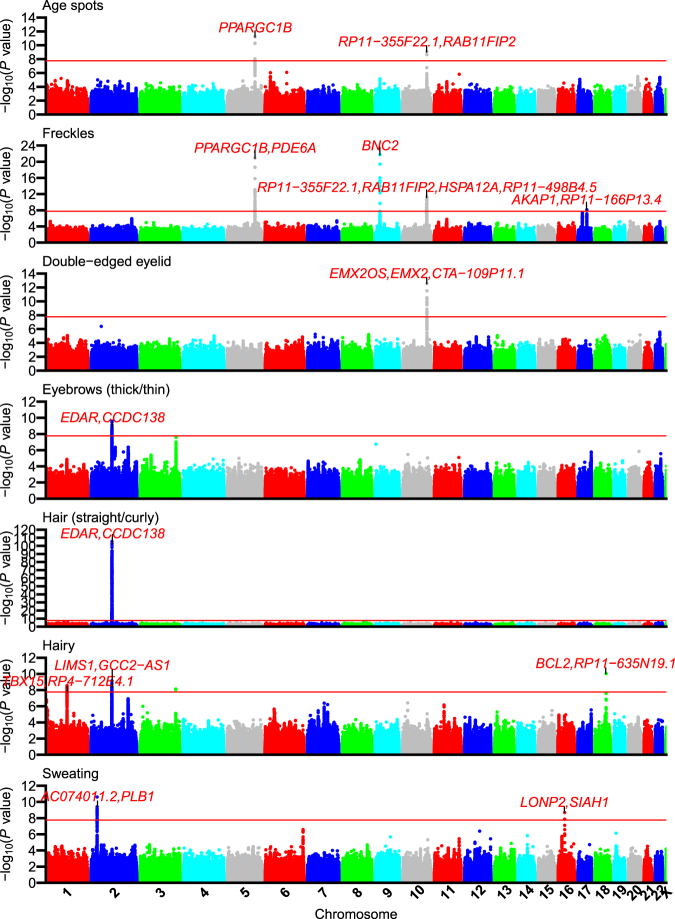
Meta-analysis Manhattan plots for skin-related phenotypes. Manhattan plots of −log_10_(P value) for skin phenotype GWAS analyses after summary statistics-based imputation using 1000 Genomes Project Phase 1 Release 3 reference data. Top association signals were labeled with up to two annotated genes from Supplementary Worksheet S1 for variants with *r*^2^ > 0.8 to the top SNP. Peaks with more than two genes overlap more than one independent association signal. The horizontal red line denotes the multiple testing corrected P-value cutoff of 1.73 × 10^−8^ that was used for identifying association signals.

We performed a genome-wide association study (GWAS) of Japanese female subjects who answered a questionnaire about various phenotypes and provided DNA for genetic analysis of those traits. The data was collected in two study-stages, termed LL01 and LL02 (LL01 = 5750, LL02 = 5628), with a detailed description of the dataset and methods used for genotyping, imputation, and annotation available in a recently published GWAS report of self-reported food reactions that used the same set of samples^17^. For the current report, we analyzed up to 11311 subjects who fulfilled quality-control (QC) criteria and provided self-reported phenotype information on the presence of age spots, freckles, double-edged eyelids, eyebrow thickness (thick vs. thin eyebrows), hair morphology (straight vs. curly hair), excessive hairiness, and excessive sweating (Supplementary Table S1; plot of principal component analysis in Supplementary Fig. S1). For several phenotypes (double-edged eyelid, eyebrow thickness, hair morphology, and excessive sweating), trait data was only available from the LL02 study-stage. Meta-analysis of the two GWAS stages was performed using 536506 QC+ variants from a custom Affymetrix Axiom array, for which there was negligible inflation of genome-wide statistics observed across the seven phenotypes (λ_GC_: 1.0031–1.043; Supplementary Fig. S2). A summary-statistics based approach^18^ was used to impute genome-wide missing genotypes in order to produce the Manhattan plot (Fig. 1) and to allow for analysis of data from previous reports, but for the purposes of the association study analyses, we defined associated genomic regions as those with genotyped SNPs that achieved a multiple-testing adjusted *P* < 1.73 × 10^−8^ (see Methods). Within each associated region, we then performed genotype-based imputation^19–21^ and step-wise conditioning on top SNPs to identify independent secondary signals (*P*_*conditioned*_ < 1 × 10^−5^). Across the seven skin-related phenotypes, we identified twelve separate loci made up of sixteen independent association signals. Fifteen signals were novel associations for the current phenotype being examined (Table 1). The following sections will describe each of the signals in the context of functional annotations and eQTL signals (see Methods) in order to help identify candidate causal variants and target genes.

**Table 1:** Skin phenotype GWAS significant locus summary.

Chr.	Signal range (*r*^*2*^ > 0.5)	Sig.	rsID	Alleles	AF	LL01 *P*	LL02 *P*	Meta. *P*	Meta. OR[CI]	Genes
**Age spots and Freckles (shared)**										
5	149.19–149.23 Mb	1	rs251468	T/C	0.18	1.4 × 10^−5^	8.2 × 10^−8^	6.2 × 10^−12^	0.77[0.72–0.83]	*PPARGC1B*
10	119.58–119.60 Mb	1	rs61866017	T/G	0.16	4.6 × 10^−4^	3.2 × 10^−7^	9.7 × 10^−10^	0.78[0.73–0.85]	*RAB11FIP2*
10	119.56–119.58 Mb	2	rs35563099	T/C	0.087	8.0 × 10^−4^	9.7 × 10^−7^	1.2 × 10^−6^	0.78[0.70–0.86]	*RAB11FIP2*
**Freckles**										
5	149.19–149.23 Mb	1	rs251468	T/C	0.18	2.1 × 10^−9^	5.3 × 10^−14^	1.3 × 10^−21^	0.69[0.64–0.75]	*PPARGC1B*
9	16.79–16.81 Mb	1	rs10810635	T/C	0.47	3.5 × 10^−12^	9.4 × 10^−12^	2.1 × 10^−22^	0.76[0.72–0.80]	*BNC2, RP11–62F24.2* ^*^
10	119.56–119.58 Mb	1	rs10444039	A/C	0.088	5.0 × 10^−11^	1.8 × 10^−11^	5.6 × 10^−21^	0.60[0.54–0.67]	*RAB11FIP2*
10	118.45–118.48 Mb	2	rs12259842	T/C	0.25	3.0 × 10^−7^	7.8 × 10^−5^	7.1 × 10^−11^	1.23[1.16–1.31]	*HSPA12A* ^***^
10	119.55–119.58 Mb	3	rs10886142	T/C	0.49	2.5 × 10^−1^	6.3 × 10^−4^	1.2 × 10^−9^	1.20[1.13–1.27]	*RAB11FIP2*
10	119.58–119.60 Mb	4	rs4752116	T/C	0.21	2.1 × 10^−8^	4.2 × 10^−5^	3.3 × 10^–9^	0.81[0.76–0.87]	*RAB11FIP2*
17	55.21–55.25 Mb	1	rs17833789	C/A	0.45	9.3 × 10^−6^	5.6 × 10^−5^	2.2 × 10^−9^	1.18[1.12–1.25]	*AKAP1, MSI2*
**Double-edged eyelids**										
10	119.25–119.32 Mb	1	rs12570134	G/T	0.27	NA	8.2 × 10^−15^	8.2 × 10^−15^	0.71[0.65–0.77]	*EMX2* ^**^ *, EMX2OS* ^***^
10	119.33–119.35 Mb	2	rs1415425	C/A	0.48	NA	3.1 × 10^−5^	1.9 × 10^−7^	0.81[0.75–0.88]	*EMX2, EMX2OS*
**Eyebrows (thick/thin)**										
2	108.93–109.57 Mb	1	rs3827760	A/G	0.2	NA	1.7 × 10^−9^	1.7 × 10^−9^	0.71[0.63–0.79]	*EDAR*
**Hair (straight/curly)**										
2	108.93–109.57 Mb	1	rs260643	G/A	0.2	NA	1.6 × 10^−103^	1.6 × 10^−103^	0.30[0.27–0.33]	*EDAR*
**Excessive hairiness**										
1	119.45–119.77 Mb	1	rs984225	G/A	0.38	6.6 × 10^−7^	2.6 × 10^−3^	1.6 × 10^−8^	1.18[1.12–1.25]	*TBX15* ^***^
2	108.93–109.57 Mb	1	rs6542772	C/T	0.17	3.7 × 10^−5^	1.4 × 10^−5^	2.2 × 10^−9^	1.27[1.17–1.37]	*GCC2* ^***^ *, LIMS1-AS1* ^***^ *, LIMS1* ^**^
18	60.92–60.94 Mb	1	rs7226979	C/T	0.43	4.4 × 10^−5^	2.8 × 10^−7^	7.3 × 10^−11^	1.21[1.14–1.28]	*BCL2* ^***^
**Excessive sweating**										
2	28.83–29.05 Mb	1	rs56089836	G/C	0.42	NA	1.7 × 10^−11^	1.7 × 10^−11^	1.40[1.27–1.54]	*PPP1CB* ^***^
2	28.82–28.85 Mb	2	rs1534480	T/C	0.14	NA	3.3 × 10^−7^	7.7 × 10^−6^	1.38[1.20–1.60]	*PLB1* ^***^
16	48.26–48.45 Mb	1	rs6500380	G/A	0.14	NA	6.8 × 10^−10^	6.8 × 10^−10^	1.61[1.38–1.87]	*ABCC11*

“Sig.” refers to sequential number of an independent signal that was identified by conditioning in a genomic region. Gene/signal combinations are marked if they were had support from both ABF (*PP*_*H4.ABF*_ > 0.3). and SMR (*P*_*SMR*_ < 0.05) tests by the amount of support from the ABF test of colocalization: ^***^PP_*H4.ABF*_ > 0.9 (Strong support), ^**^PP_*H4.ABF*_ > 0.5 (Moderate support), ^*^PP_*H4.ABF*_ > 0.3 (Nominal support). “Alleles” column lists “Effect-allele/Other-allele”. Effect-allele is oriented to the minor allele across all subjects’ genotypes.

### Age spots and freckles association signals

Previously reported freckles/facial pigmented spots association signals identified in two GWAS studies using Northern European ancestry samples^11,12^ displayed only weak evidence for replication in our Japanese dataset (Table 2; Supplementary Table S2). Both studies’ top SNPs in the basonuclin 2 gene (*BNC2*) locus were only nominally significant in our data (*P*_rs2153271_ = 0.0044; *P*_rs62543565_ = 0.0351), and in the interferon regulatory factor 4 (*IRF4*), melanocortin 1 receptor (*MC1R*), and agouti signaling protein *(ASIP*) loci, their top SNPs were predominantly monomorphic in 1000G East Asian samples (Supplementary Table S2) or were only weakly significant in our data (Table 2; *P*_*IRF4,rs9405675*_ = 0.0113; *P*_*MC1R:rs8060934*_ = 0.0027). Two previous reports^12,22^ had also constructed a compound heterozygosity score using six *MC1R* missense variants: rs1805005: Val60Leu; rs1805007: Arg151Gly; rs1805008: Arg160Trp; rs1805009: Asp294His; rs2228479: Val92Met; rs885479: Arg163Gln. The first four listed SNPs were monomorphic in Japanese, and one of the two polymorphic variants was nominally associated with freckles in our dataset (*P*_rs2228479_ = 0.00044; *P*_rs885479_ = 0.61053), but neither was associated with age-spots.

**Table 2:** Known skin-related phenotype associations with FDR < 0.1 in current study.

Report author	Reported phenotype	Current study phenotype	Chr.	rsID	Pos.	Gene	EAS AF	EUR AF	Reported *P*	Current study P	Current study FDR
Eriksson	Freckles	Freckles	6	rs9328192	434364	IRF4	0.36	0.46	4.1E-10	0.015	0.097
Eriksson	Freckles	Freckles	6	rs9405675	444600	IRF4	0.36	0.63	1.9E-09	0.011	0.091
Eriksson	Freckles	Freckles	9	rs2153271	16864521	BNC2	0.23	0.58	4.0E-10	4.4E-03	0.091
Eriksson	Freckles	Freckles	16	rs8060934	89920025	MC1R	0.73	0.55	1.4E-09	2.7E-03	0.091
Eriksson	Freckles	Freckles	16	rs8049897	90024202	MC1R	0.27	0.13	1.6E-30	5.1E-03	0.091
Jacobs	Freckles	Freckles	16	rs8051733	90024206	MC1R	0.28	0.28	3.1E-09	0.012	0.091
Jacobs	Freckles	Freckles	16	rs62052243	90026152	MC1R	0.28	0.28	1.4E-09	0.011	0.091
Jacobs	Freckles	Freckles	16	rs8063761	90027626	MC1R	0.28	0.28	8.1E-10	9.4E-03	0.091
Adhikari	Hair shape	Hair morph.	2	rs3827760	109513601	EDAR	0.87	0.01	3.0E-119	3.1E-103	2.8E-102
Adhikari	Beard thick.	Hairiness	1	rs11121667	11038476	C1orf127	0.29	0.24	3.8E-07	2.9E-06	2.0E-05
Adhikari	Eyebrows	Eyebrows	2	rs3827760	109513601	EDAR	0.87	0.01	1.2E-07	1.7E-09	3.4E-08
Adhikari	Eyebrows	Hairiness	2	rs3827760	109513601	EDAR	0.87	0.01	1.2E-07	5.1E-09	5.1E-08
Adhikari	Unibrow	Eyebrows	2	rs3827760	109513601	EDAR	0.87	0.01	1.5E-07	1.7E-09	7.8E-08
Pickrell	Unibrow	Eyebrows	2	rs3827760	109513601	EDAR	0.87	0.01	2.4E-15	1.7E-09	7.8E-08
Adhikari	Unibrow	Hairiness	2	rs3827760	109513601	EDAR	0.87	0.01	1.5E-07	5.1E-09	1.2E-07
Pickrell	Unibrow	Hairiness	2	rs3827760	109513601	EDAR	0.87	0.01	2.4E-15	5.1E-09	1.2E-07
Pickrell	Unibrow	Hairiness	3	rs1345417	181511951	SOX2–ATP11B	0.29	0.63	1.7E-31	7.6E-09	1.4E-07
Pickrell	Unibrow	Eyebrows	3	rs1345417	181511951	SOX2–ATP11B	0.29	0.63	1.7E-31	9.5E-06	1.2E-04
Pickrell	Unibrow	Eyebrows	5	rs4476718	124075470	ZNF608	0.87	0.81	2.2E-14	7.9E-03	0.066
Pickrell	Unibrow	Hairiness	10	rs113334738	18271858	SLC39A12	0.02	0.15	4.3E-09	9.1E-03	0.070
Pickrell	Unibrow	Eyebrows	10	rs7905367	54334653	DKK1–MBL2	0.06	0.77	4.4E-15	6.0E-04	6.1E-03
Pickrell	Unibrow	Hairiness	10	rs7905367	54334653	DKK1–MBL2	0.06	0.77	4.4E-15	5.6E-03	0.051
Pickrell	Unibrow	Hairiness	11	rs66716358	44330610	ALX4	0.51	0.47	7.6E-13	5.9E-06	9.1E-05
Pickrell	Unibrow	Eyebrows	11	rs66716358	44330610	ALX4	0.51	0.47	7.6E-13	3.3E-04	3.8E-03

Previously reported associations were extracted for SNPs associated with freckles from Supplementary Table 1 of Jacobs *et al*.^12^, Table 2 of Eriksson *et al*.^11^, and Table 1 of Motokawa *et al*.^46^. Unibrow phenotype data came from the Strongest Associations table from 2016 Pickrell *et al*.^13^, and hair related data from Table 1 and Supplementary Table 8 of 2016 Adhikari *et al*.^13^, merged with 1000 Genomes Project site information (EAS and EUR allele frequencies), and with the current study’s summary statistics based genome-wide imputed data for freckles, hair morphology, hairiness, or eyebrow thickness phenotypes.

In the current study, we further identified five loci associated with age-spots and/or freckles (Table 1). Two were associated with both phenotypes at genome-wide significance, and three were genome-wide significant only for the freckles phenotype (Fig. 1). To examine whether variants in those GWAS signals had evidence as regulatory variants, we performed colocalization analysis of GTEx Portal^23^ eQTL statistics from their single-tissue and multi-tissue Metasoft modified random effects (RE2)^24,25^ analyses using the Approximate Bayes Factor (ABF)^26^ and Summary-data based Mendelian Randomization (SMR)^27^ methods (see Methods). GWAS/eQTL signal pairs that were at least nominally significant by the SMR test-of-linkage (*P*_*SMR*_ < 0.05), non-significant for SMR’s HEIDI test-of-pleiotropy (HEIDI: heterogeneity in dependent instruments; *P*_*HEIDI*_ ≥ 0.05) and had posterior probability of colocalization from the ABF test *PP*_*H4.ABF*_ > 0.3 were considered as having nominal evidence for colocalization, while pairs with *PP*_*H4.ABF*_ > 0.5 and *PP*_*H4.ABF*_ > 0.9 were considered as having moderate and strong support, respectively. Lower values of *P*_*SMR*_ and higher values of *P*_*HEIDI*_ also provide stronger evidence of colocalization/pleiotropy of causal variants at a GWAS and an eQTL signal.

High LD SNPs in the top chr9:16.79–16.81 Mb locus (rs10810635; *P*_*freckles*_ = 2.09 × 10^−22^; Supplementary Fig. S3a) overlapped introns of *BNC2*, with some overlapping RoadMap Epigenomics^28,29^ predicted enhancer activity as well as DNase hypersensitive sites (DHS) and Transcription factor binding sites (TFBS) identified in the ReMap 2018 database^30,31^ 70 kb upstream of an alternative promoter (Supplementary Worksheet S3; Supplementary Fig. S3c). Visualization of the 25-state chromatin state model in 127 reference epigenomes also found that for three SNPs (rs10810635, rs67920508, rs16935073) active enhancer function was predicted in skin-spots relevant tissue samples such as foreskin fibroblasts and foreskin melanocytes (http://egg2.wustl.edu/roadmap/web_portal/imputed.html#chr_imp). The colocalization tests showed nominal support for antisense RNA *RP11-62F24.2* eQTLs in only a single-tissue (Supplementary Fig. S3b). That ncRNA overlaps the alternative *BNC2* promoter, suggesting that these SNPs may indirectly regulate *BNC2* expression in a tissue or cell-type restricted manner.

The age-spots/freckles signal in the Peroxisome proliferator-activated receptor gamma coactivator 1 beta (*PPARGC1B*) gene at chr5:149.19–149.23 Mb (Supplementary Fig. S4; Supplementary Worksheet S1; top SNP rs251468; *P*_*age spots*_ = 6.24 × 10^−12^; *P*_*freckles*_ = 1.27 × 10^−21^) had four of five high LD (*r*^*2*^_*equiv*_ > 0.8; Supplementary Worksheet S3) freckles variants overlapping predicted enhancer function, and the top variant had function predicted in twelve tissue/cell types while also overlapping TFBS for multiple TFs. No SNPs were eQTLs in GTEx Portal data, so the functional impact of these SNPs on *PPARGC1B* could not be confirmed from *in silico* analysis.

The chr10:119.55–119.60 Mb region downstream of the RAB11 family interacting protein 2 (*RAB11FIP2*) contained three independent freckles and two independent age-spots signals (Supplementary Figs S5a–c and S6a,b; Table 1). We observed overlap between high LD variants in these signals and predicted epigenetic activity (Supplementary Figs S5d and S6c; Supplementary Worksheets S2 and S3), but there was no indication from eQTL data that linked putative regulatory function to target genes.

**Figure 2:**
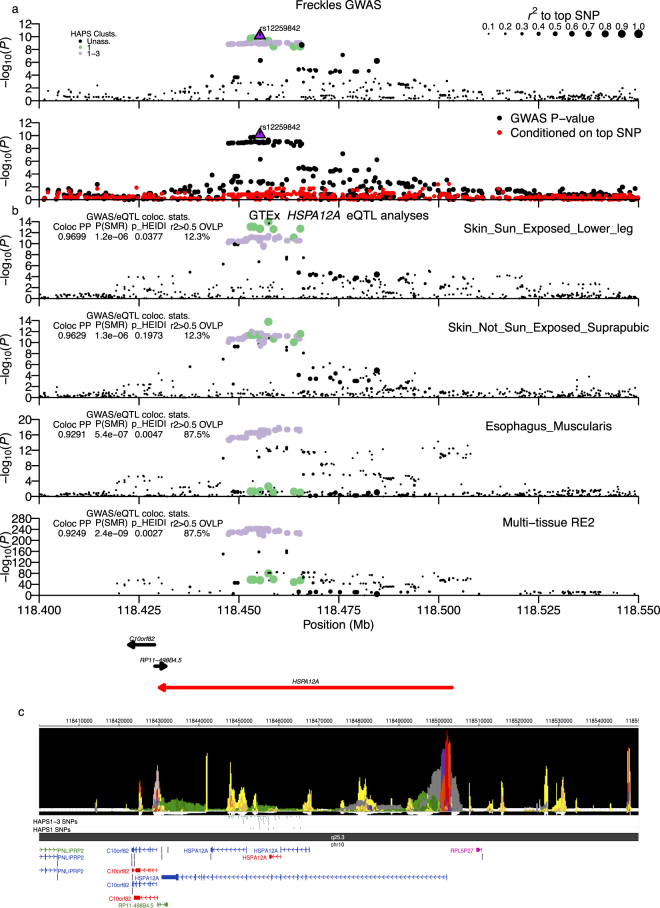
Chr10:118.45–118.48 Mb (*HSPA12A*) freckles locus. Freckles associated SNPs possess strong evidence for colocalization with single-tissue and multi-tissue *HSPA12A* eQTL signals. (**a**) Regional association plots of −log_10_(P-values) around the Chr10:118.45–118.48 Mb (*HSPA12A*) freckles association locus. Top sub-panel presents points sized by *r*^*2*^ to the top GWAS SNP and coloured either black or by haplotype cluster HAPS1 or HAPS1-3 assignment (legend in the upper left corner). The bottom sub-panel shows SNPs with and without conditioning on the top SNP. (**b**) Shows −log_10_(P-values) for *HSPA12A* eQTL data for single-tissue and multi-tissue Metasoft RE2 analyses, with labels at the right-hand side. Points are sized and coloured the same as in the top (**a**) sub-panel. Colocalization statistics from ABF and SMR methods and the percent of mod. LD GWAS SNPs overlapping mod. LD eQTL SNPs are shown at the left of each sub-panel as an inlayed table. GENCODE gene models are shown below the eQTL plots. (**c**) presents output from the WashU EpiGenome Browser of an epilogos plot of the Roadmap Epigenomics 25-state imputed model of epigenetic states along with tracks of high LD candidate causal variants divided by assigned haplotype cluster(s) and GENCODE transcript models in the region. All panels are plotted on the same x-axis coordinates.

A nearby unlinked freckles association signal at chr10:118.45–118.48 Mb overlapped the heat shock protein family A (Hsp70) member 12A (*HSPA12A*) gene (Fig. 2a; top SNP rs12259842; *P* = 7.08 × 10^−11^). We observed two groups of moderate/high LD SNPs based on effect-allele frequency (EAF ~0.34 vs ~0.25, respectively) and haplotype cluster analysis. The two haplotype clusters (HAPS1 and HAPS3) overlapped the effect-allele (set to minor allele in Japanese) at those SNPs in combination or by HAPS1 alone (Supplementary Fig. S7). HAPS1 versus HAPS1-3 SNPs showed only slight differences in the association statistics (*P*_*HAPS1*_ ~ 2 × 10^−10^ vs *P*_*HAPS1-3*_ ~ 8 × 10^−10^). Similarly, they clustered together as strongly associated with *HSPA12A* expression in the two freckles pertinent GTEx skin tissue samples (Fig. 2b), but only HAPS1-3 SNPs were strongly significant eQTLs in non-skin tissue samples (bottom panels of Fig. 2b; Supplementary Fig. S8b). Both ABF and the SMR test-of-linkage were strongly supportive of colocalization of GWAS and eQTL signals, but HEIDI was only non-significant (supporting pleiotropy) in the sun un-exposed skin tissue, for which both HAPS1 and HAPS1-3 SNPs clustered closest together. In epigenetics data, four peaks overlapped the GWAS SNPs, with all four overlapping HAPS1-3 SNPs and two overlapping HAPS1 SNPs (Fig. 2c; Supplementary Worksheet S3; approx. peak centers: peak #1 = 118.448 Mb, peak #2 = 118.451 Mb, peak #3 = 118.454 Mb, and peak #4 = 118.458 Mb). In peak #1, two HAPS1-3 SNPs (rs1900500, rs2921967), overlapped promoter and enhancer function predicted in large numbers of tissue/cell types. In a 1 kb region of peak #2, enhancer function was predicted in a larger number of tissue/cell types (from 60–70 tissue/cell types) for seven HAPS1-3 variants, while in peak #3, HAPS1-3 SNP rs2907231 overlapped enhancer elements identified in forty tissue/cell types and lay in a region bound by forty TFs. Given that HAPS1 eQTLs were restricted to skin tissue samples, we expected a causal regulatory SNP to have epigenetic function predicted only in a restricted set of tissues that included skin cell types. Of the top five GWAS HAPS1 SNPs, two did not overlap any predicted epigenetic function and one SNP overlapped enhancer function predicted in a large number of tissues, although skin tissue samples were not among them. However, the other two top HAPS1 SNPs (rs3010484, rs2907229), which lay in peak #4, had enhancer function predicted in only a limited number of tissue types (skin, brain) including one foreskin melanocytes tissue sample. Those two SNPs were also the strongest eQTLs in both GTEx skin tissues, resided right next to each other, and appeared to be perfectly linked with each other in each 1000 G population based on allele frequencies (Supplementary Worksheet S3). Our results suggest that at least three HAPS1-3 variants regulate *HSPA12A* expression in a multi-tissue fashion, while a pair of HAPS1 SNPs have regulatory activity at least partially restricted to skin tissues.

The final signal (top SNP rs17833789; *P*_*freckles*_ = 2.19 × 10^−9^) lay 2–3 kb downstream of the A-kinase anchoring protein 1 (*AKAP1*) gene and 7–10 kb upstream of the musashi RNA binding protein 2 (*MSI2*) gene in the chr17:55.21−55.25 Mb region (Supplementary Fig. S9a). High LD SNPs overlapped enhancer function predicted in only a small number of tissue samples (Supplementary Fig. S9b; Supplementary Worksheet S3), and enhancer activity for three of those (rs17833789, rs62060349, rs9907841) was only identified in melanocyte skin tissue samples. No SNPs were eQTLs for either *AKAP1* or *MSI2*, but analysis of FANTOM5 CAGE expression data^32,33^ pointed to a functional difference for *MSI2* compared to *AKAP1. AKAP1* displayed similar RLE expression values in keratinocytes and both dark and light-skin subjects’ melanocytes (*mean*_*Keratinocytes*_ = 17.6; *mean*_*dark.melanocytes*_ = 9.1; *mean*_*light.melanocytes*_ = 14.0). In contrast, *MSI2* exhibited low expression levels in both keratinocytes and dark skin melanocytes (*mean*_*Keratinocytes*_ = 3.8; *mean*_*dark.melanocytes*_ = 4.5), but values in light-skin subjects’ melanocytes were approximately six-times higher (*mean*_*light.melanocytes*_ = 27.8).

The seven top SNPs across the skin-spots loci accounted for 5.2% of freckles phenotypic variance (calculated as a “pseudo-R2”), while the three age-spots signals explained 1.36% of its variance (Supplementary Table S5). We observed a large degree of overlap between the two phenotypes (Supplementary Table S6), with 47.8% (3553/7438) of age-spots cases also being freckles cases, and 88.1% (3553/4034) of freckles cases being age-spots cases. We constructed a genetic risk score (GRS) from the regression model coefficients and found that the proportion of freckles (any versus strong freckling) increased linearly with respect to GRS bin (Supplementary Fig. S10).

### Double eyelid association signals

**Figure 3:**
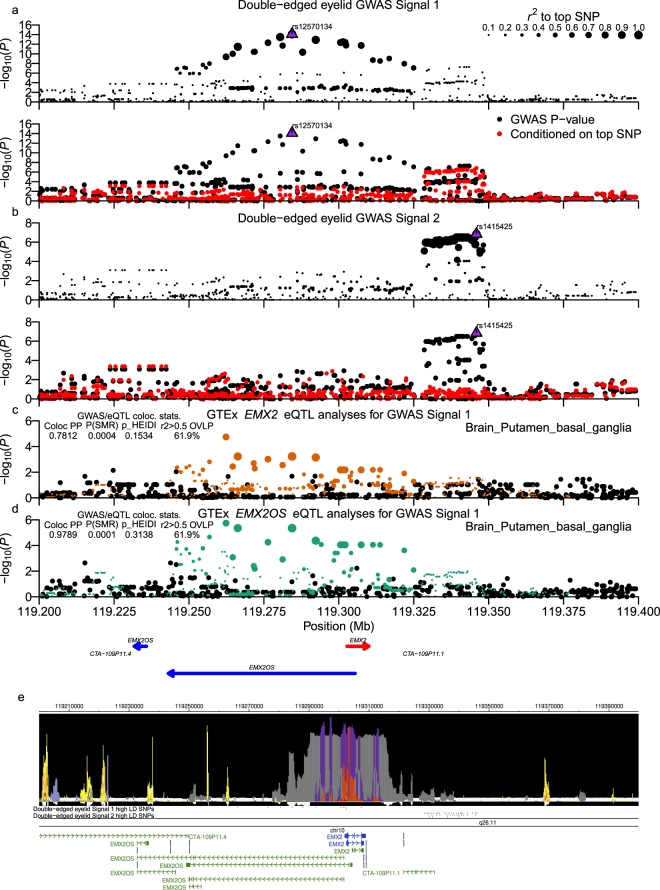
Chr10:119.25–119.32 Mb (*EMX2*/*EMX2OS2*) double-edged eyelid locus. Two independent signals were identified in the chr10:119.25–119.32 (*EMX*/*EMX2OS2*) double-edged eyelid association locus, and Signal 1 SNPs displayed moderate and strong evidence for colocalization with eQTL signals for *EMX2* and the anti-sense RNA gene *EMX2OS*, respectively. (**a**,**b**) Present regional association plots of −log_10_(P-values) for Signal 1 and 2, respectively. (**c**,**d**) Show −log_10_(P-values) from Brain_Putamen_basal_ganglia tissue samples for association with *EMX2* and *EMX2OS* expression, respectively. GWAS and eQTL panels are configured as described in the Fig. 2 legend, but eQTL panel point colours denote different independent eQTL signals, with the top signals in a region in green and orange. GENCODE gene models are shown below the eQTL plots. (**e**) Presents output from the WashU EpiGenome Browser of an epilogos plot of the Roadmap Epigenomics 25-state imputed model of epigenetic states along with tracks of high LD signal 1 and signal 2 variants and GENCODE transcript models in the region.

The chr10:119.25–119.35 Mb locus contained two novel independent signals associated with the double eyelid phenotype (Fig. 3a,b; Supplementary Worksheet S4), and SNPs in both signals (Signal #1: chr10:119.25–119.32 Mb: rs12570134: *P* = 8.15 × 10^−15^; Signal #2: chr10:119.33–119.35 Mb: rs1415425: *P*_*cond*._ = 1.90 × 10^−7^) were within or near the empty spiracles homeobox 2 (*EMX2*) gene or the antisense long non-coding RNA (lncRNA) gene *EMX2OS*. Two signal #1 SNPs overlapped predicted promoter activity (rs12777466, rs12777755: Supplementary Worksheet S4), were conserved in both GERP and SiPhy annotations, lay within the *EMX2* 5′-UTR, and both were predicted in HaploReg to cause a TF motif change. Notably, promoter activity was predicted in tissues pertinent to facial development^34,35^, such as human embryonic stem cell (ESC) derived CD56+ mesoderm cells. Signal #2 displayed weak functional evidence, with only two of sixty-five high LD intergenic SNPs overlapping any epigenetic annotation (Fig. 3e). GWAS/eQTL analysis using brain putamen (basal ganglia) tissue samples identified moderate to strong support for colocalization/pleiotropy of signal #1 with *EMX2* (Fig. 3c) and *EMX2OS* eQTLs (Fig. 3d), respectively.

### Replication analysis of previous hair-related trait associations

We looked for replication/overlap with known signals involving similar hair-related traits. We mapped previously analyzed GWAS phenotypes to one from the current study (phenotype mapping of previous = current phenotypes: hair shape = hair morphology; eyebrow thickness, unibrow, or beard thickness = excessive hairiness; eyebrow thickness or unibrow = eyebrow thickness) and extracted any SNPs achieving *FDR* < 0.1 (Table 2). The strongest replication was for a previous association with hair shape in the *EDAR* gene (Table 2). However, in general, previous associations displayed little overlap with our results. None of four strong associations with beard thickness (*P* < 5 × 10^−8^) were validated in our excessive hairiness data, but one of four suggestive associations achieved *P* = 2.9 × 10^−6^ in our data (rs11121667 in *C1orf127*; *P*_*reported*_ = 3.8 × 10^−7^; Table 2 and Supplementary Table S3a)^36^. Only one of ten previous eyebrow thickness associated SNPs was validated in our eyebrow thickness and excessive hairiness analyses (rs3827760; *P*_*reported*_ = 1.2 × 10^−7^; Table 2 and Supplementary Table S3b,c), while 19 of 57 SNPs previously associated with unibrow were low frequency/monomorphic in Japanese or not present in 1000G, and only one of seven suggestive and six of 50 strong unibrow associations could be replicated in our data. Five of the replicated unibrow signals were associated with both eyebrow thickness and hairiness traits (Table 2; Supplementary Table S3d,e). The strongest of the non-*EDAR* signals that replicated lay in the chromosome 3 *SOX2*-*ATP11B* gene region, and our top hairiness SNP in the region matched that reported by Pickrell *et al*.^13^ for unibrow (*P*_*rs1345417*_ = 7.57 × 10^−9^).

### Pan-hair related phenotype association signals at chr2:108.93–109.57 Mb

For the three hair-related traits (hair morphology, eyebrow thickness, and excessive hairiness), we identified overlapping association signals in and around the *EDAR* gene on chromosome 2 (Supplementary Fig. S11a–c). The eyebrow thickness and hair morphology associations almost completely shared high LD SNPs (Supplementary Worksheets S5 and S6), and a known missense variant (rs3827760) that has been associated with multiple traits^3,4,37–40^ ranked first and second across the two signals, respectively (rs3827760: *P*_*eyebrows*_ = 1.71 × 10^−9^; *P*_*straight hair*_ = 3.14 × 10^−103^). As noted above, our results replicate a previously reported association between that variant and straight vs. curly hair in Asian population samples^37^ (Table 2; Supplementary Tables S3 and S4).

In contrast, top imputed excessive hairiness variants in the chr2:108.93–109.57 Mb locus (*r*^*2*^ > 0.8: n = 230; top SNP rs6542772: *P* = 2.16 × 10^−9^; Supplementary Worksheet S7) were only moderately linked to rs3827760 (*r*^*2*^ = 0.507) and did not overlap *EDAR*. Rather, they resided within or near the LIM zinc finger domain containing 1 (*LIMS1*), the GRIP and coiled-coil domain-containing protein 2 (*GCC2*), or RAN binding protein 2 (*RANBP2*) genes (Supplementary Fig. S11d). Many of those variants were GTExPortal eQTLs for *LIMS1* and *GCC2* as well as the antisense RNAs *LIMS1-AS1* (AC010095.5) and *GCC2-AS1* (AC012487.2) (Supplementary Worksheets S7). Colocalization analysis identified strong support for *LIMS1-AS1* in multi-tissue RE2 data (Supplementary Fig. S12b) and for GCC2 in a single tissue (Supplementary Fig. S12d), although the HEIDI tests did not support pleiotropy with GCC2. Moderate support for colocalization/pleiotropy was also found for *GCC2-AS1* in both single-tissue and RE2 analyses (Supplementary Fig. S12c) and for *LIMS1* in several tissues (Supplementary Fig. S12e; Supplementary Worksheet S11,S12); only *LIMS1* eQTLs were in very high LD (*r*^*2*^ > 0.9) to both GWAS and eQTL top variants.

### Hair density related traits associated loci

In addition, we identified two novel association signals for the excessive hairiness trait in the chr1:119.45–119.77 Mb and chr18:60.92–60.94 Mb regions.

**Figure 4:**
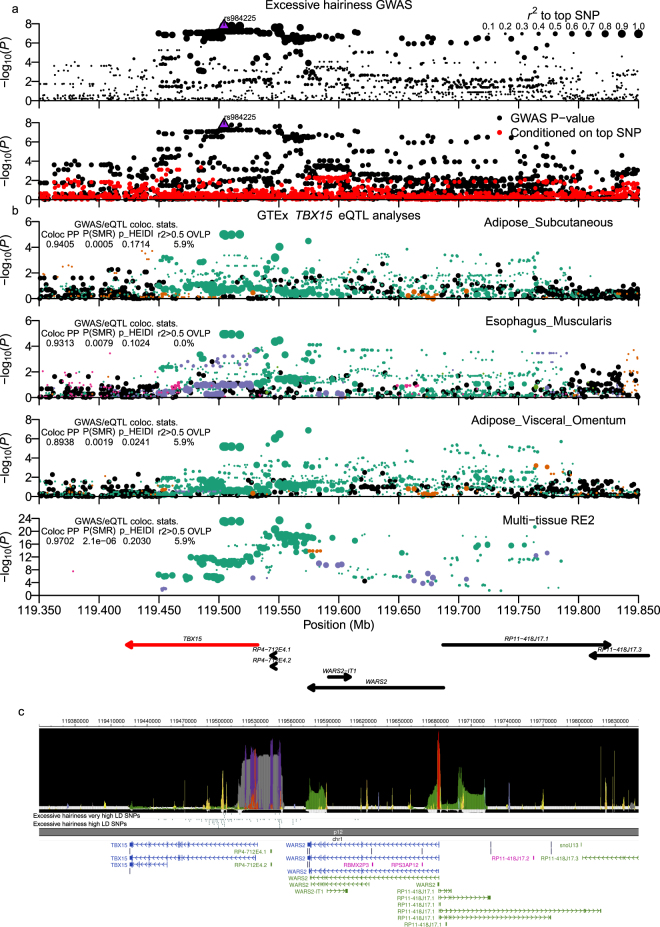
Chr1:119.45–119.77 Mb (*TBX15*/*WARS2*) excessive hairiness locus. Excessive hairiness associated SNPs in the Chr1:119.45–119.77 Mb locus possessed strong evidence for colocalization with single-tissue and multi-tissue *TBX15* eQTL signals. (**a**) Shows regional association plots of −log_10_(P-values). (**b**) Shows −log_10_(P-values) for *TBX15* eQTL data for single-tissue and multi-tissue Metasoft RE2 analyses. GWAS and eQTL panels are configured as described in the Figs 2 and 3 legends. (**c**) Presents output from the WashU EpiGenome Browser of an epilogos plot of the Roadmap Epigenomics 25-state imputed model of epigenetic states along with tracks of high LD (*r*^*2*^_*equiv*_ > 0.8) and very high LD (*r*^*2*^_*equiv*_ > 0.9) variants and GENCODE transcript models in the region.

Chr1:119.45–119.77 Mb locus (Fig. 4a) SNPs lay within the T-box 15 (*TBX15*) gene or between *TBX15* and the Tryptophanyl-tRNA synthetase, mitochondrial (*WARS2*) gene (Supplementary Worksheet S7); overlap with promoter and intronic enhancer annotations within *TBX15* (Fig. 4c) supported it being the functional gene. Although very high LD GWAS variants (*r*^*2*^ > 0.9) were strong eQTLs for *WARS2* expression in multiple GTExPortal tissues (Supplementary Worksheet S7), they were actually lowly ranked within *WARS2* multi-tissue eQTL statistics. In contrast, colocalization/pleiotropy analysis identified strong support for *TBX15* eQTLs across several tissues as well as multi-tissue RE2 data (Fig. 4b).

**Figure 5:**
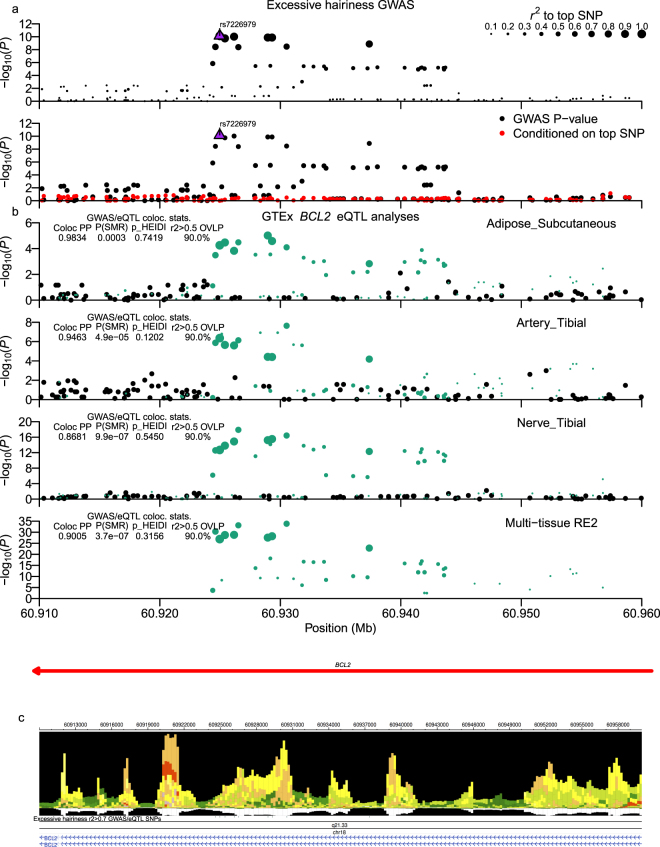
Chr18:60.92–60.94 Mb (*BCL2*) excessive hairiness locus. Excessive hairiness associated SNPs in the Chr18:60.92–60.94 Mb (*BCL2*) locus possess strong evidence for colocalization with single-tissue and multi-tissue *BCL2* eQTL signals. (**a**) Regional association plots of −log_10_(P-values) around the Chr18:60.92–60.94 (*BCL2*) excessive hairiness locus. (**b**) Shows −log_10_(P-values) for *BCL2* eQTL data for single-tissue and multi-tissue Metasoft RE2 P-values. GWAS and eQTL panels are configured as described in the Figs 2 and 3 legends. (**c**) Presents output from the WashU EpiGenome Browser of an epilogos plot of the Roadmap Epigenomics 25-state imputed model of epigenetic states along with a track of variants with mod./high LD in both GWAS and eQTL data (*r*^*2*^ > 0.7 in GWAS and eQTL analyses for subcutaneous adipose, tibial artery, and tibial nerve tissues) and GENCODE transcript models in the region.

In the final excessive hairiness locus at chr18:60.92−60.94 Mb (Fig. 5a; rs7226979; *P* = 7.26 × 10^−11^), all top linked SNPs lay within *BCL2* gene introns and overlapped predicted enhancer activity (Supplementary Worksheet S7; Fig. 5c). Colocalization/pleiotropy of GWAS and *BCL2* eQTL variants was strongly supported by both methods in multiple tissues (Fig. 5b; Supplementary Worksheet S7).

### Excessive sweating association signals

For the excessive sweating phenotype, we identified two novel loci, with one locus at chr2:28.82−29.05 Mb containing two independent association signals and another locus with a single signal at chr16:48.26−48.45 Mb.

**Figure 6:**
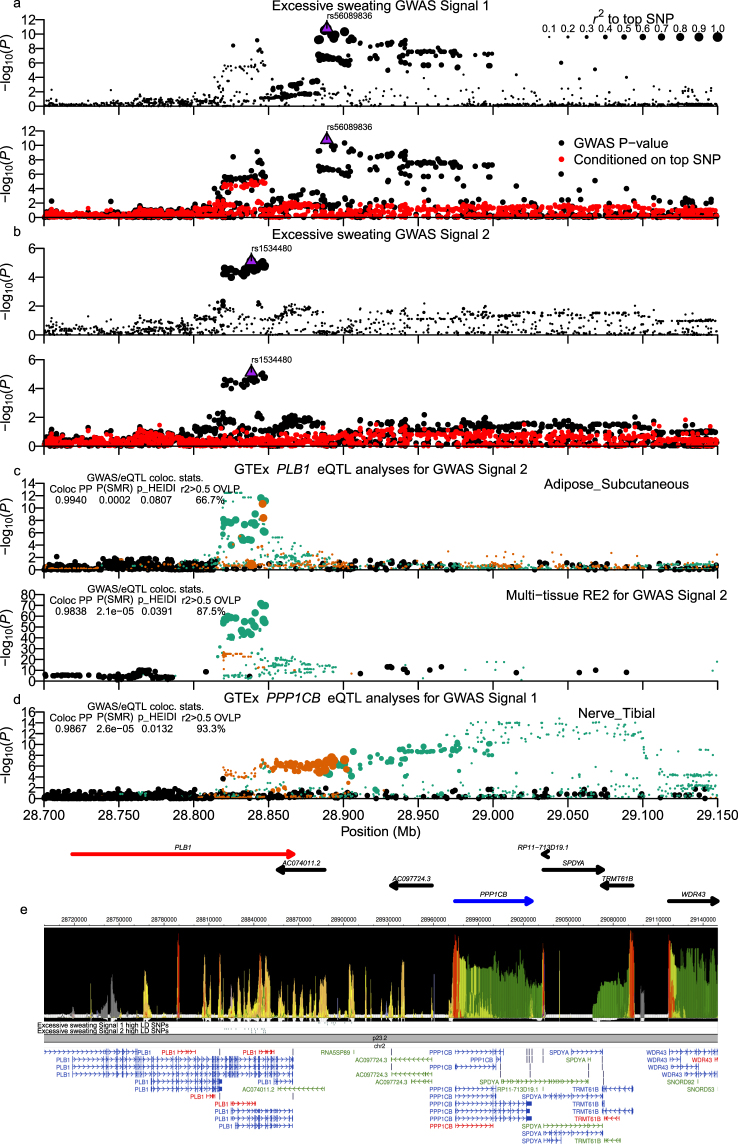
Chr2:28.82–29.05 Mb (*PLB1*/*PPP1CB*) excessive sweating locus. Of two independent GWAS signals in the chr2:28.82–29.05 Mb (*PLB1*/*PPP1CB*) excessive sweating locus, Signal #1 colocalizes with *PPP1CB* eQTL from a single tissue, while Signal #2 colocalizes with a *PLB1* eQTL signal identified in multiple tissues. (**a**,**b**) Show regional association plots of the Chr2:28.82–29.05 Mb (*PLB1*/*PPP1CB*) locus for the two independent signals. (**c**) Colocalization analyses of Signal #2 SNPs with *PLB1* subcutaneous adipose tissue and multi-tissue Metasoft RE2 eQTLs. (**d**) Colocalization analysis of Signal #1 SNPs with *PPP1CB* tibial nerve eQTLs. GWAS and eQTL panels are configured as described in the Figs 2 and 3 legends. (**e**) Presents output from the WashU EpiGenome Browser of an epilogos plot of the Roadmap Epigenomics 25-state imputed model of epigenetic states along with tracks of high LD signal 1 and signal 2 variants and GENCODE transcript models in the region.

The chr2 region’s primary association signal (Signal #1: chr2:28.83−29.05 Mb; top SNP rs56089836: *P* = 1.70 × 10^−11^) lay upstream of the protein phosphatase 1 catalytic subunit beta (*PPP1CB*) gene and downstream of the Phospholipase B1 (*PLB1*) gene, while the secondary association signal (signal #2: chr2:28.82–28.85 Mb; top SNP rs1534480: *P*_*unconditioned*_ = 3.28 × 10^−7^; *P*_*Cond. SNP 1*_ = 7.74 × 10^−6^) resided within the *PLB1* gene (Fig. 6a,b; Supplementary Worksheet S8). Both signals had variants that overlapped predicted enhancer and/or promoter elements (Fig. 6e). One missense SNP was found in the second signal (rs7601771; H/D; Cac/Gac) but was predicted by all dbNSFP^41^ consequence predictions in the UCSC Genome Browser^42^ to be tolerated/benign. Colocalization/pleiotropy analysis identified strong support for overlap of signal #2 variants with *PLB1* eQTLs from multiple tissues (Fig. 6c and Supplementary Fig. 13e), with *AC097724.3* multi-tissue eQTL data (Supplementary Fig. 13d), and with *AC074011.2* eQTLs from a single-tissue sample (Supplementary Fig. S13c). A secondary *PPP1CB* eQTL signal in tibial nerve tissue had strong support for colocalization with Signal #1 variants (Supplementary Fig. S13f) in ABF and basic SMR tests but had no support of pleiotropy from the HEIDI test.

Chr16:48.26−48.45 Mb locus variants (Supplementary Fig. S14a; top SNP rs6500380; *P* = 6.84 × 10^−10^; Supplementary Worksheet S8) lay within or near the Lon peptidase 2, peroxisomal (*LONP2*), ATP binding cassette subfamily C member 11 (*ABCC11*), and siah E3 ubiquitin protein ligase 1 (*SIAH1*) genes. One high LD SNP was a known *ABCC11* missense variant (rs17822931; *P* = 2.02 × 10^−9^; *ABCC11* c.538 G > A (p.Gly180Arg)). All linked variants were also *LONP2* eQTLs in multiple tissues (Supplementary Worksheet S8), but no significant colocalization with eQTLs was identified. Our results suggest the likely causal variant in this locus is the known coding SNP, rs17822931.

## Discussion

In this study, we performed GWAS analyses of seven skin-related phenotypes (age spots, freckles, single vs. double eyelids, hairiness, straight vs. curly hair, eyebrow thickness, and excessive sweating) and identified twelve loci containing sixteen association signals, of which fifteen were novel discoveries. The single previously reported association signal involved the association of hair morphology phenotypes (straight vs. curly hair, eyebrow thickness) with SNPs in *EDAR* (top SNPs rs260643 and rs3827760, respectively). The following will briefly discuss our findings for each of these phenotypes and place the putative causal genes in the context of current biological and medical knowledge.

### Skin-spot phenotypes

Japanese women suffer in early adulthood from pigmented spots, followed by facial wrinkles in their thirties, forties, and fifties^2^. Freckles, which are pigmented macules generally between 1–3 mm in diameter, characteristically occur on the face as well as the back of the hands, shoulders, and neck^43^. Age spots (lentigines), which are a notable outward manifestation of skin aging, have been reported to occur earlier and be more conspicuous in skin of Asians compared to that of Caucasians^44^. It is well known that pigmented spots are associated with chronic ultraviolet exposure^45^, but the genetics related to this phenotype has been less well understood, especially for individuals of Asian ancestry. Motokawa *et al*. first reported that *MC1R* (melanocortin-1-receptor) gene variants were associated in Asians with melanogenic phenotypes and thus identified both environmental factors and genetic factors as involved in the production of age spots and freckles^46^. Two previous reports’ top SNPs in the *IRF4* (rs12203592), *MC1R* (rs12931267, rs35063026), and *RALY*/*ASIP* (rs619865, rs6059655) regions were monomorphic in 1000G ASN and had *MAF* <= 0.01 in 1000G AFR samples, and therefore likely represent population specific causal variants^11,12^. Our study identified a novel association signal in the known *BNC2* gene locus and found novel loci in and around the genes *PPARGC1B*, *RAB11FIP2*, *HSPA12A*, and *AKAP1*/*MSI2*.

BNC2 is a zinc-finger transcription factor that appears to be necessary for development of craniofacial and long bones as well as ectodermal appendages (hair follicles, salivary glands, palatal rugae)^47,48^, and it has previously been associated with skin pigmentation and pigmented spots^11,12,49^. Epigenetic and eQTL data indicate the most likely function for the signal’s variants are as an enhancer for an alternative promoter near *BNC2* exon 3^10^. *PPARGC1B* codes the Peroxisome proliferator-activated receptor gamma coactivator 1-beta, the target of which (PPARγ) has previously been associated with melanogenesis. Various earlier reports support this gene’s involvement in pigmentation related phenotypes, with 2,4,6-Octatrienoic acid reported to act via PPARγ activation to promote melanogenesis and antioxidant defense in melanocytes^50^, and ciglitazone, a PPARγ agonist, observed to cause an increase of melanin production in cells and cultured skin^51^. Our analysis suggests that variants at this locus may regulate *PPARGC1B* expression through modification of enhancer elements. *RAB11FIP2* codes for the Rab11 family-interacting protein 2, which acts in regulating vesicular transport from the endosomal recycling compartment to the plasma membrane (http://www.uniprot.org/uniprot/Q7L804). Depletion of Rab11 causes accumulation of pigment in melanocytes^52^, the Rab11b isoform mediates melanin exocytosis from melanocytes^53^, and interaction between RAB11FIP2 and myosin 5b (MYO5B) was reported to regulate movement of vesicles containing RAB11a^54^. In the *AKAP1*/*MSI2* locus, both genes possess support for putative function, but support for *MSI2* appears strongest. Response elements for another novel skin spot associated gene, *PPARGC1B*, have been reported to help regulate *AKAP1* expression^55^, but we found *MSI2* to be differentially expressed in light-skin melanocytes versus dark-skin melanocytes and keratinocytes from FANTOM5. MSI2 was also previously found necessary to maintain the resting state of hair follicle stem cells^56^, and the hair follicle bulge is also a main location of melanocytic stem cells^57^.

In contrast to those four loci, for which SNPs lacked eQTL activity for protein-coding genes, skin-spots variants around *HSPA12A* were associated with gene expression. *HSPA12A* is a member of the 70-kDa heat shock protein (HSP70) family, which is a highly-conserved family of chaperone genes that help facilitate many aspects of proteostasis such as protein folding, multi-protein complex assembly, and transport^58^. HSPA12A’s sub-cellular compartment has been localized to exosomes in urine samples^59,60^ and specifically localized to exosomes that express markers of podocytes, which are similar to the filopodia that form between melanocytes and keratinocytes^61^. That suggests that HSPA12A may play a role in the transfer of melanin from/along melanocytic filipodia.

To understand how our top skin-spots associations related to skin-pigmentation phenotypes, we examined overlap with GWAS summary statistics for two UK Biobank (UKBB)^62^ variables (Ease of skin tanning, Skin colour) for which GWAS analyses were performed by the Gene ATLAS (http://geneatlas.roslin.ed.ac.uk; see Methods)^63–65^. Across the seven primary and secondary skin-spot signals’ top SNPs, we observed co-localization with top skin-pigmentation SNPs for *PPARGC1B* chr5:149.19–149.23 Mb (rs251468: *P*_*freckles*_ = 1.27 × 10^−21^, *P*_*tanning*_ = 3.32 × 10^−50^, *P*_*skin colour*_ = 2.52 × 10^−41^) and *RAB11FIP2* chr10:119.56–119.58 Mb (rs10444039: *P*_*freckles*_ = 5.62 × 10^−21^, *P*_*tanning*_ = 3.78 × 10^−71^, *P*_*skin colour*_ = 4.93 × 10^−53^). That suggests that overall skin-pigmentation and acquisition of pigmented spots at least partially share mechanisms for their development.

In addition, the overlap between age-spots and freckles cases/controls in our sample suggests that the definition/translation of age-spots and freckles in Japan (shimi and sobakasu) may differ from the United States and European sense, with freckles (sobakasu) in Japan perhaps representing more of an extreme end of a continuum of skin spot accumulation due to sun exposure. Based on a genetic risk score analysis (Supplementary Fig. S10), we found that the presence of multiple effect-alleles imparts a greater pre-disposition for acquisition of freckles. Further GRS analysis in independent cohorts would be needed to confirm that, and in the future, as direct to consumer genetic testing becomes more prevalent, it may be warranted to recommend to individuals with multiple effect alleles for these association signals that they reduce sun exposure to avoid the appearance of pigmented skin spots.

### Double versus single eyelid phenotype

The human eyelid is a complex craniofacial structure consisting of interconnecting and layered substructures made up mostly of skin, adipose, muscular, and nerve tissues that help protect the eye from injury and insult due to external environmental threats such as temperature and humidity variation, wind, and dust and airborne objects^66^. The structure and appearance of the human upper eyelid shows variation in the amount of upper eyelid crease, with individuals having some degree of upper eyelid crease or those who lack such a crease termed double and single eyelids (or mono-lids), respectively^67^. In Asians, individuals with either single eyelids or eyelids with intermediate features make up approximately 44% of the population^68^, but they are very uncommon in most other ethnic populations^67^. Such eyelid variation has been ascribed to anatomical differences for the location at which the orbital septum fuses with the levator aponeurosis^69^, as well as to underlying differences in the shape and structure of the orbital socket^70^. Because individuals who possess double eyelids are sometimes considered more attractive compared to those having single eyelids, double eyelid surgery (blepharoplasty) has become a popular medical procedure^67,71,72^, with more than 40,000 Japanese women receiving eyelid modification surgery every year.

In this study, we identified double eyelid associated SNPs that were also eQTLs for *EMX2* and the antisense RNA *EMX2OS*, the latter of which has been reported to regulate *EMX2* expression^73,74^. *EMX2* is a homeobox-containing transcription factor gene that appears important in development of skeletal and neural structures^75–77^. In light of recent reports of *EMX2* in the context of craniofacial development^35^, our results suggest that these SNPs may regulate *EMX2* expression at embryonic stages important for human facial structure development that lead to concomitant upper eyelid differences.

### Hair morphology related phenotypes

Some women are affected by the presence of dense eyebrows, which may necessitate additional time spent on processing their eyebrows. In addition, scalp and body hair can assume different morphological shapes, both in terms of shaft thickness and shaft straightness/curl. In our study, the only genome-wide significant signals associated with dense eyebrows or straight/curly hair phenotypes replicated a known association of the *EDAR* missense variant rs3827760^3,4,36,37,78^. *EDAR* codes for the ectodysplasin A receptor, which is a membrane-bound receptor of the TNF-R family, mutations of which have also been linked with Mendelian diseases associated with skin such as Ectodermal dysplasia^79–81^ as well as with facial and ear characteristics^38,39^, and dental traits^40^.

### Excessive hairiness (hirsutism) phenotype

Excessive hairiness, which is medically termed hirsutism, represents excess hair growth in females, and can generally be divided into sub-types corresponding to: (1) individuals with high circulating androgen levels or high sensitivity to androgen, or (2) idiopathic hirsutism, for which an individual possesses normal androgen levels and no other discernible cause for the excess hair growth^82^. From examination of previous hair-density related trait analyses, we identified overlap with both eyebrow thickness or hirsutism signals (Table 2), which suggests some shared mechanisms between these phenotypes. In addition to validation of SNPs associated with these previous traits, our study identified three novel hirsutism loci that were also eQTLs for *BCL2*, *GCC2* and *LIMS1*, and *TBX15*.

BCL2 acts as an anti-apoptotic regulatory protein that blocks cell death and has a known role in the cycle of life-and-death related to hair follicle growth^83^. Since regulation of catagen (apoptosis-driven involution of hair follicles) stage timing is an extremely important part of the process^84^, our analysis suggests that differences in *BCL2* expression due to variants at this locus may shift the timing or length of catagen and thereby lead to variation in hair-density between individuals.

In the *GCC2*/*LIMS1* locus, our analysis suggests that SNPs may impact expression of both genes. GCC2 localizes to the trans-Golgi network and functions in the tethering and capture of inbound vesicles in endosomal transport^85–87^. LIMS1 localizes to focal adhesion plaques to regulate cell adhesion and spreading through interaction with integrin-linked kinase (ILK)^88–90^ and Parvins as part of the ILK, PINCH, and Parvin complex (IPP)^91^. ILK was reported to be necessary for hair morphogenesis^92^, and deletion of *LIMS1* from mouse keratinocytes was shown to impair hair follicle growth^93^. In addition, LIMS1 contributes to BCL2-dependent survival signaling and acts to inhibit JNK-mediated apoptosis^94^.

TBX15 is known to be involved in development, particularly in chondrocyte hypertrophy and skeletal limb development^95^, and Tbx15 was previously shown to be involved in hair pigmentation and hair length in mice^96^. It has also been related to the determination of muscle fiber-type^97^ and metabolic subtypes of adipocytes^98,99^. One of our signal’s top variants (rs984222) was previously associated with BMI and Waist-hip ratio (WHR) in individuals of European ancestry^100^, which was later confirmed in a trans-ethnic meta-analysis of European and African-American ancestry population samples^101^. Using the Gene ATLAS Region PheWAS tool, we also identified rs984222 as positively associated with WHR (β = 0.00147, *P* = 6.95 × 10^−26^), but while it was also associated with whole-body/leg/arm bioimpedance measurements (β_*whole body*_ = 1.2651, *P*_*whole body*_ = 3.29 × 10^−21^; β_*leg(left)*_ = 0.56755, *P*_*leg(left)*_ = 3.79 × 10^−18^; β_*arm(left)*_ = 0.6744, *P*_*arm(left)*_ = 1.57 × 10^−17^; β_*arm(right)*_ = 0.63848, *P*_*arm(right)*_ = 1.64 × 10^−16^; β_*leg(right)*_ = 0.52175, *P*_*leg(right)*_ = 1.58 × 10^−15^), it was not significantly associated with Body fat percentage (β = −0.0030593 *P* = 0.81055). In addition, it was positively associated with Standing height (β = 0.070321, *P* = 1.05 × 10^−9^) but negatively associated with BMI (β = −0.04843, *P* = 5.00 × 10^−7^) and Hip circumference (β = −0.081466, *P* = 1.52 × 10^−5^). For our analysis, we had included BMI as a covariate (Supplementary Table S1), suggesting that the association with excessive hairiness was also not related to adiposity. Taken together, these findings suggest that these *TBX15* regulating variants act in a pleiotropic manner that impacts skeletal development, fat store distribution, and hair follicle density and/or activity.

### Excessive sweating phenotype

Sweating ability plays an important function in regulating body temperature and is performed by eccrine and apocrine sweat glands present on the skin. Eccrine glands secrete a clear fluid made up mostly of water, NaCl, and other salts, and compared to apocrine glands, they are present in the greatest number across the human body (~2–4 million) and provide for most of sweat’s cooling properties^102^. Eccrine gland secretion is mainly controlled through cholinergic and to a lesser extent adrenergic signaling originating from the pre-optic region of the hypothalamus, but other signaling molecules appear to act at a local level^103^.

Hyperhidrosis, the clinical term for excessive sweating, can be caused by various pathologies^104,105^, and the presence of increased sweating can lead to embarrassment in social situations and sometimes impairs quality of life (QOL)^106^. In the absence of known causes, idiopathic hyperhidrosis does not appear to be a result of morphological changes in eccrine gland numbers or size but rather a complex dysfunction of autonomic nervous system central control^107^. In studies of hyperhidrosis patients, reports have identified other autonomic nervous system abnormalities, such as differences for cardiovascular stress responses, that point to over-functioning of sympathetic^108^ and possibly parasympathetic nervous system fibers^109^. Previously, a genome-wide linkage analysis of primary palmar hyperhidrosis in Japanese identified a significant region at 14q11.2-q13, but that region did not show significant associations in our analysis^110^. In the current study of excessive sweating, we found associations with SNPs in two gene regions on chromosome 2 and chromosome 16.

In the chromosome 2 *PLB1*/*PPP1CB* region, our analyses identified associated variants in two independent signals as either *PLB1* or *PPP1CB* eQTLs. Previous reports lend support for both *PLB1* and *PPP1CB* as plausible genes for the association with sweating. *PLB1* codes for phospholipase B1 protein (PLB), which is an enzyme that has both phospholipase A1 and A2 enzymatic activities. *PLB1* was originally identified in humans by its expression in the epidermis and suggested to function by promoting the skin barrier function by breakdown of lipids into free fatty acids^111^. In addition, based on its role in promoting acrosome exocytosis in sperm^112–114^, there is a possibility that it could function to modulate secretory processes in other situations such as sweating. On the other hand, *PPP1CB* encodes the beta-subunit of the Serine/threonine-protein phosphatase PP1, which is a key enzyme involved in the regulation of a large number of cellular processes^115^. Based on previous reports that phosphorylation of the water-specific channel aquaporin-5 (AQP5) regulates its ability to mediate water flow^116,117^ we hypothesize that *PPP1CB* eQTL SNPs may effect sweat production by modulating the amount of PPP1CB that is present and thereby influencing phosphorylation levels of AQP5 or other proteins necessary for sweat gland function.

The single signal on chromosome 16 encompassed a region including *ABCC11* and *LONP2* genes. After eQTL and LD structure analysis, we concluded that a known missense SNP in *ABCC11* (rs17822931) was the likely causal variant among the five tightly linked SNPs in this locus. *ABCC11* encodes ATP binding cassette subfamily C member 11, which is a member of the multi-drug resistance protein (MRP) sub-family of the ATP-binding cassette gene family. The missense SNP has previously been associated with dry versus wet earwax types^118^ and axillary ozmidrosis (body odor)^119–121^. The current study is the first report that shows that this SNP is also associated with hyperhidrosis.

## Conclusions

In this report, we identified a dozen loci encompassing 16 association signals for the dermatological phenotypes of skin-spots, double vs. single eyelids, eyebrows (thick/thin), hair (straight/curly), excessive hairiness, and excessive sweating. For skin-spot phenotypes (freckles and age-spots), we found four novel loci encompassing the *PPARGC1B*, *RAB11FIP2*, *HSPA12A*, and *AKAP1/MSI2* genes, along with a novel East Asian signal in the known *BNC2* gene locus. Future research should combine these signals with association signals identified in other ethnic groups and confirm the preliminary genetic risk score that we calculated using the current dataset. Such a score may help women identify their risk for skin-spot acquisition and take appropriate actions to mediate their impact. For the double eyelid phenotype, *EMX2* represents the likely regulatory target of the two independent signals, but understanding their actual functional impact will require analyzing the variants’ effect on *EMX2OS* and *EMX2* expression in the context of particular developmental stages. For hair-related phenotypes, we replicated previous reports that implicated the rs3827760 *EDAR* missense variant with hair morphology and hair straightness/curliness, but additionally, we identified that SNPs near *EDAR* are associated with excessive hairiness (hair density) and are eQTLs for two neighboring protein-coding genes, namely *GCC2* and *LIMS1*, as well as for neighboring lncRNAs. The LD structure in the *EDAR* region in Japanese makes it difficult to confirm the findings in further Japanese population samples, so future research should examine the relationship of these eQTL variants with the trait in other population samples. In contrast to the *EDAR* missense variant rs3827760, these high LD SNPs are common alleles in other world-wide population samples, and therefore, they should be amenable to validation. For the excessive sweating phenotype (hyperhidrosis), previous GWAS analyses have not been performed, and our current analysis implicated variants in one region that may regulate *PLB1* and/or *PPP1CB* expression, and in another region, identified a known missense variant in *ABCC11*. An excessive rate of sweating may affect QOL in an individual’s social life, and therefore, cosmetic procedures to reduce hyperhidrosis has demand in the medical beauty-care industry. Further experimental research about these genes in the context of excessive sweating will be necessary and will hopefully lay a foundation for identifying systemic or topical agents to ameliorate their effects.

## Methods

Details about the subject, sample, and phenotype data collection, sample processing and genotype Quality Control (QC) procedures, statistical analysis, linkage disequilibrium (LD) statistics calculation, *in silico* functional annotation of variants, and eQTL analyses can be found in a recent report that used the same underlying set of samples^17^. The following sections will include brief versions of the fully described methods, methods that were not included in that report, or for which previous methods differ (different procedures or upgraded datasets) or require further explanation.

### Subject, sample, and phenotype data collection

Subjects were voluntarily enrolled in a study investigating the genetics of various human traits run by the MTI (http://www.mti.co.jp/eng/) subsidiary EverGene. Questionnaires soliciting trait information were filled-out by subjects online. Subjects were collected in two stages, denoted as LL01 and LL02, with 11378 individuals completing the questionnaires and providing DNA samples (LL01 = 5750, LL02 = 5628). The Institutional Review Board at the Tsukuba International Clinical Pharmacology Clinic approved the study design, such as the consent form, general questionnaire topics, and genotyping, and the study was performed in accordance with applicable regulations and guidelines. Informed consent was obtained from each patient for sample collection, genotyping, trait questionnaire, and trait analysis using genome-wide association study analysis.

### Sample processing, genotyping and quality control

There were 607857 total variants assayed using the custom Axiom array EverGene1 chip. For downstream analyses, the following QC criteria had to be fulfilled in both stages: 1) ≥99% call-rate, 2) MAF ≥ 0.01, 3) HWE P-value ≥ 1 × 10^−6^, and 4) concordance-rate >90%. After QC, there were 536506 variants.

### Principal component analysis (PCA)

We performed principal component analysis (PCA)^122^ with PLINK2 v1.90pVer.b3.42 (release date 16 Aug 2016)^123,124^ to identify population structure. A PCA plot of samples used in the current analysis is shown in Supplementary Fig. S1, while a complete description of the PCA based process for sample filtering can be seen in Supplementary Fig. S1 of the earlier Khor SS, *et al*. report^17^.

### Identification of duplicated samples

Using LD-pruned data, we ran identify-by-descent (IBD) analysis with PLINK2 ver. 1.90p’s and filtered data as previously described. For the current study, there were 11311 subjects who passed QC procedures and who also answered at least some of the skin phenotype questions on the questionnaire. After running the current GWAS analysis, we checked and found that a very small number of sample pairs (n = 14) were identified with PI_HAT > 0.1875 and PI_HAT < 0.8, generally suggestive of second to first-degree relatives within the sample-set. Since the percent of total samples in that PI_HAT range was very low (~0.12%), their presence should have little impact on the overall GWAS statistics.

### Definition of skin phenotype cases and controls

Certain constitutional phenotypes were asked as the question “Please describe your constitution, ease, strength, etc. [with respect to certain possible traits]”). The possible answers corresponded to the English phrases “Very applicable”, “Slightly true”, and “Not applicable”. For these traits, cases were considered as those answering “Very applicable” or “Slightly true” and controls as those answering “Not applicable”. For this paper, the traits included age spots (Japanese = Shimi), freckles (Japanese = Sobakasu), and hairiness (Japanese = Kebukasa).

Another set of constitutional questions were queried in the form “For each of the following, are you closer to A or B? Please answer subjectively.” Phenotypes queried in such a manner included thick vs. thin eyebrows, straight vs. curly hair, and excessive sweating. For thick/thin eyebrows, responses were A: Thick eyebrows (Japanese: Mayuge ga koi) and B: Thin eyebrows (Japanese: Mayuge ga usui). For straight/curly hair, responses were A: Straight hair (Japanese: Kami ga sutorēto) and B: Curly hair (Japanese: Kami ga kusekke). For excessive sweating, responses were A: It is easy to sweat (Japanese: Ase o kaki yasui) and B: It is hard to sweat (Japanese: Ase o kaki nikui). For each of those phenotypes, A responders were set as cases and B responders set as controls.

The final constitutional question was a multiple-choice question that asked “Please choose all that apply to yourself from the following (Multiple selections possible)”. One possible choice was for the double-edged eyelid phenotype (Japanese: Futaemabuta). Subjects checking that box were considered as responding affirmatively to the presence of the phenotype and thus used as cases, while those that did not mark the selection were considered as responding negatively to the presence of the phenotype and thus were used as controls.

### Statistical analysis and genotype imputation

The R 3.4.1 statistical environment was used for management of data, statistical analyses, and figure plotting^125^. The primary association analyses for LL01 or LL02 datasets was run using PLINK2’s logistic regression analysis method (-logistic flag). For each phenotype analyzed, we included PC1 and PC2 (from the stage 3 PCA described above) as covariates, and then Age or BMI as covariates if a test of them against the phenotype in a regression model showed that they were significant (P < 0.05). The specific covariates that were included in each regression analysis are shown in Supplementary Table S1. Meta-analysis statistics combined LL01 and LL02 data using the inverse-variance weighting method with beta-coefficients and standard errors from the regression analyses^126^. Our assumption for the effective number of SNPs (M_E_) for the current genotyping platform was previously described^17^, resulting in a single GWAS *P*-value cut-off of *P*_*meta*_ < 1.21 × 10^−7^ (0.05/411,521). Based on the number of phenotypes analysed in the current analysis, we defined primary association signals as those with genotyped variants that achieved a multiple-testing adjusted *P*-value cut-off of *P*_*meta*_ < 1.73 × 10^−8^ (*P*_*meta*_ < 1.21 × 10^−7^/7 skin phenotypes).

For plotting the Manhattan plot and to allow comparison/validation with data from previous reports, we performed a genome-wide summary statistics based imputation of the meta-analysis data using the program DISTMIX^18^ and the 1000 Genomes Phase 1 Release 3 reference data^127^. For primary association signals, we analyzed imputed genotype data that we produced using a more accurate genotype-based imputation method that was described in the Khor *et al*. report. Variants were filtered for allelic R2 > 0.7. In each region of imputed data, we performed step-wise logistic regression analysis conditioning on the top imputed variant in each signal until no further variants with *P* < 1 × 10^−5^ were identified; top variants achieving that significance level after a conditioning step were considered as secondary association signals.

Allele frequencies and LD statistics were calculated using PLINK 1.9 and then imported into R for analysis. For variance explained calculations, we imported the genotype data into R and used generalized linear model (glm) for logistic (family = ‘binomial’) or linear (family = ‘gaussian’) regression analysis using all of the top SNPs with PC1, PC2, and Age or BMI as covariates in a full regression model of the phenotype of interest. Using the R *rms* package^128^, we performed a likelihood ratio test of the full model (glm1) against the model without the associated variants (glm0) and then calculated a pseudo-R2^129^ as a surrogate for the proportion of variance explained. With n being the number of samples and LR the χ^2^ statistic from the likelihood ratio test comparison of glm1 and glm0 (lrtest(glm0, glm1)), we calculated pseudo-R2 as (1 − exp(−LR/n))/(1 − exp(2logLik(glm0)/n)).

For the freckles case-control phenotype, we calculated a genetic risk score (GRS) using the riskScore function from the R package PredictABEL^130^ with extracted components from the glm models. Within each GRS value, we then summarized the proportion of individuals who were positive for either any freckles (Freckles = Very applicable or Slightly applicable) or strong freckles (Freckles = Very applicable).

### LD measures

We used PLINK 1.9 to calculate traditional LD *r*^*2*^ and *D*’ measures from the imputed genotype data. Additionally, we calculated an alternative measure that we term *r*^*2*^_*equiv*_, which is based on conditional regression analysis to measure the signal decrease at a SNP B relative to a top SNP A and was described in detail in the Khor *et al*. report. Moderate LD SNPs were generally considered as those with *r*^*2*^_*equiv*_ > 0.5 and high LD SNPs as those with *r*^*2*^_*equiv*_ > 0.8.

### *In silico* functional analysis of associated variants

We attempted to use the current dbSNP147 rsID as much as possible. The RsMergeArch.bcp.gz table was imported into R from NCBI’s ftp site and we identified the current rsID for SNPs present in the various annotation sources used below.

We annotated genotyped and 1000G variants using HaploReg 4.1^131^, including dbSNP gene function annotation and evolutionary conservation scores (GERP), and GWAS and eQTL results from the Genome-Wide Repository of Associations Between SNPs and Phenotypes (GRASP)^132^. Since HaploReg and 1000G used different dbSNP versions, we identified both the current rsID and all previously used rsIDs for each SNP, passed all rsIDs to HaploReg for annotation, and then processed the output to resolve the current rsID with the one actually used by HaploReg.

Since HaploReg is no longer maintained or updated, we added/updated data from several sources. A current version of the NHGRI/EBI GWAS Catalog was downloaded on February 6, 2018 from the UCSC Genome Browser and used for annotation of SNPs for previous GWAS results^133,134^. For more comprehensive eQTL associations, we downloaded multi-tissue eQTL Metasoft results (file: GTEx_Analysis_v7.metasoft.txt.gz) from the GTExPortal dataset page (https://www.gtexportal.org/home/datasets) along with files for variant and gene meta-information (variants: GTEx_Analysis_2016-01-15_v7_WholeGenomeSeq_635Ind_PASS_AB02_GQ20_HETX_MISS15_PLINKQC.lookup_table.txt.gz, genes: gencode.v19.genes.v7.patched_contigs.gtf)^135^. That data was imported into R and used to annotate SNPs with P-values from multi-tissue analysis using fixed-effects (FE), random effects (RE), and Metasoft modified random effects (RE2) models. That data was also used in the colocalization analysis that is described in the next section. We also labelled each SNP in our output with the minimum single-tissue P-value along with a text string of any gene/tissue pairs and their P-values that surpassed a multiple testing corrected 0.05/#tissues. Overlap of variants in each associated region with experimentally defined transcription factor binding sites (TFBS) was done using the ReMap 2018 annotation tool^30,31^.

In addition, we summarized the RoadMap Epigenomics data using our own scripts. We downloaded the 25-state/12-mark imputed chromosome segment model of epigenetic states from http://egg2.wustl.edu/roadmap/data/byFileType/chromhmmSegmentations/ChmmModels/compressedStateTracks/hg19_chromHMM_imputed25.gz, and we used bedtools (command flags “intersect -wb -a”) to extract segment state data for genomic regions associated in the GWAS. We then added meta-information for samples (https://docs.google.com/spreadsheet/ccc?key=0Am6FxqAtrFDwdHU1UC13ZUxKYy1XVEJPUzV6MEtQOXc&usp=sharing#gid=15) and information about imputed marks (http://egg2.wustl.edu/roadmap/data/byFileType/chromhmmSegmentations/ChmmModels/imputed12marks/jointModel/final/annotation_25_imputed12marks.txt) to be able to aggregate across tissue samples from the same anatomical class and collapse information from similar epigenetic states. Epigenetic state counts shown in Supplementary Worksheets S1–S8 summarize Promoter overlap counts from four Promoter states (TssA, PromU, PromD1, Prom D2), Enhancer overlap from ten states (TxReg, TxEnh5′, TxEnh3′, TxEnhW, EnhA1, EnhA2, EnhAF, EnhW1, EnhW2, EnhAc), Transcription overlap from four states (Tx5′, Tx, Tx3′, TxWk, TxReg), and bivalent/poised from PromP and PromBiv states. Other reported states only had a single state in the dataset.

Variant overlap with coding gene models was done using the UCSC Genome Browser GENCODE Ver. 24 tracks (wgEncodeGencodeBasicV24lift37.txt.gz and wgEncodeGencodeAttrsV24lift37.txt.gz)^42,136^, with gene transcripts merged to identify gene coding start and stop coordinates, and then overlaps with SNPs identified using the R Bioconductor GenomicRanges packages. We labeled SNPs with four categories of genic overlap/nearness: 1) “within” = SNP between start and stop coordinates of a gene’s coding region, 2) “upstream” = SNP < 100 kb upstream of the gene start position, 3) “downstream” = SNP < 40 kb downstream of the gene stop position, 4) “closest” = for SNPs with no genes fulfilling the first three rules, we picked the closest gene to the SNP. “Closest” gene is not provided as a separate column but listed in a column “Genes (all)” that either contains the union of within/upstream/downstream genes for a SNP, or if those are missing, contains the closest gene. The 100 kb upstream and 40 kb downstream cutoffs were chosen based on previous reports that analyzed the general distance from Transcription Start Site (TSS) and Transcription End Site (TES) within which most eQTL SNPs were identified^137,138^.

### Analysis of colocalization of GWAS and eQTL signals

For colocalization testing of GWAS and GTEx eQTL association signals, we used the Approximate Bayes Factor (ABF) method in the R *coloc* (ver. 2.3–7) package’s coloc.abf function^26^ and the Summary data-based Mendelian Randomization method from the SMR program (ver 0.702); SMR is available on the CNS Genomics web-site (http://cnsgenomics.com/software/smr/#Overview)^27^. For each GWAS signal, we determined a list of pertinent genes from the gene and eQTL annotations in Supplementary Worksheets S1–S8 and then checked the GTEx Portal’s Gene eQTL Visualizer to identify relevant tissues in which association with those genes was observed. For a subset of the relevant tissues, we downloaded the complete GTEx Version 7 single-tissue eQTL files found on the datasets page under “Tissue Specific All SNP Gene Associations” for those tissues and processed the data in R.

Since we observed multiple independent eQTL signals for certain genes, we first processed a locus’ single-tissue or multi-tissue data for a particular gene into groups of SNPs that were in LD to a particular unassigned top eQTL variant. Briefly, a gene’s eQTL data was sorted by association statistics, and SNPs that had LD *r*^*2*^ > 0.01 to the top unlinked SNP were assigned to that SNP. If a SNP had LD *r*^*2*^ > 0.01 but the signal was very strong compared to what one would expect based on the LD, then it was left unassigned. That was determined by calculating the proportion of signal strength at a SNP B relative to the top SNP A (−log_10_(B)/−log_10_(A)) and then dividing by the *r*^*2*^. If that value was large (>10), then it was considered that the signal at SNP B was not due to just LD and SNP B was not assigned to SNP A’s signal. LD was calculated using PLINK2 across either EUR or AFR samples’ data from 1000 Genomes Project Phase 3. Although the GTEx samples were mostly of European ancestry, it was also apparent from top SNPs’ MAFs that some signals were coming from the African ancestry data, so heuristic MAF cutoffs were used to determine which 1000G population sample should be used for the LD calculation: 1) If a top SNP had MAF_EUR_ < 0.01 and MAF_AFR_ < 0.01, then we could not calculate LD statistics and a SNP was not assigned to any signal, 2) if MAF_EUR_ < 0.01 and MAF_AFR_ ≥ 0.01, then the AFR data was used for LD calculation, 3) if MAF_EUR_ ≥ 0.01 then EUR data was used for LD. The MAF_EUR_ < 0.01 and MAF_AFR_ ≥ 0.01 SNPs were often monomorphic in EAS samples, so including putative AFR ancestry specific signals in our data was incorporated mostly to produce figures that included all eQTL variants that were visible in the GTEx Portal browser.

One important difference existed between the ABF and SMR tests in terms of data input: the coloc.abf test could use either beta-coefficients and standard errors or P-values as input, but the SMR test could use only the former. In both cases, we considered the use of beta and SE as preferable. However, while beta-coefficients and standard errors were available for single-tissue data, they were not available for the multi-tissue Metasoft RE2 analysis, which was preferable since the RE2 analysis was reported to be more powerful for identifying eQTLs that act across multiple tissues in certain instances. Therefore, to pick the most powerful choice at a particular signal, we used the FE beta and SE for multi-tissue data if the FE statistics were strongly correlated with those from the RE2 analysis, but otherwise, we used the RE2 P-values as input to the ABF test along with required MAF and sample-size values. If that was done, then we would expect that the SMR results, which had to use the FE statistics as input, would differ from the ABF results. The status for whether FE beta was used for the ABF analysis is reported in Supplementary Worksheets S9 and S10. Since GTEx eQTL analyses were performed using standardized expression values, we assumed a value of 1.0 for the standard deviation of expression trait values in the analysis using coloc.abf.

The ABF method outputs posterior probabilities for five states H0–H4: *PP*_*H0.ABF*_ = no causal variant, *PP*_*H1.ABF*_ = only trait 1 has causal variant, *PP*_*H2.ABF*_ = only trait 2 has causal variant, *PP*_*H3.ABF*_ = two different causal variants, *PP*_*H4.ABF*_ = a single common causal variant (i.e., colocalized). The SMR test outputs two P-values: one for the SMR test (*P*_*SMR*_) and the other for their HEIDI test (heterogeneity in dependent instruments; *P*_*HEIDI*_). The SMR test by itself tests for linkage between a causal GWAS variant and a causal eQTL variant but does not provide evidence that the same causal variant impacts both traits. On the other-hand, HEIDI tests for heterogeneity across linked SNPs in a region and tries to determine whether pleiotropy exists in addition to simply linkage. For the purposes of this report, we considered a GWAS/eQTL signal pair as having at least nominal support for colocalization/pleiotropy if it was nominally significant for the SMR test (*P*_*SMR*_ < 0.05), non-significant for the HEIDI test (*P*_*HEIDI*_ ≥ 0.05), and had *PP*_*H4.ABF*_ > 0.3. If a signal had *PP*_*H4.ABF*_ > 0.9 or *PP*_*H4.ABF*_ > 0.5, then a signal was described as having strong or moderate support, respectively. Additionally, lower values of *P*_*SMR*_ combined with higher *P*_*HEIDI*_ values provided more evidence for colocalization.

### Haplotype cluster analysis

For one association signal, we performed haplotype cluster analysis to examine the genetic structure between putative causal variants. For the chr10:118.45–118.48 Mb locus that was examined, we calculated the maximum effect allele frequency (*EAF*_*max*_) across linked SNPs with *r*^*2*^_*equiv*_ > 0.8 (*EAF*_*max*_ = 0.35) and extracted associated variants *EAF* > 0.01, *EAF* < = *EAF*_*max*_, *P*_*meta-analysis*_ < 1 × 10^−5^, *r*^*2*^ > 0.5, and *D’* > 0.98. Then, we determined a set of non-redundant SNPs by calculating the Canberra distance between SNPs in the imputed phased haplotypes using the R *dist* function, performed complete hierarchical clustering using the *hclust* function, and identified clusters of SNPs with less than 1% dissimilarity from one another. From each cluster, we then extracted a single exemplar SNP. To perform haplotype clustering, we then extracted haplotype data for the non-redundant set of SNPs, calculated the Euclidean distance between each haplotype, and performed complete hierarchical clustering. Labels were assigned to haplotypes using the *cutree* function with a tree height cutoff determined from examining the plotted tree and picking a height that yielded a sensible number of haplotype clusters.

### FANTOM5 CAGE peak analysis

Regional filtering of FANTOM5 CAGE data^33,139^ was performed using the ZENBU browser^140^, the CAGE peak data table downloaded, and summaries produced around appropriate sample types.

Browser link for: AKAP1 analysis: http://fantom.gsc.riken.jp/zenbu/gLyphs/#config=ONHzqgf2E5Xtmnpsh2gURB;loc=hg19::chr17:55162544.55162663.

Browser link for MSI2 analysis: http://fantom.gsc.riken.jp/zenbu/gLyphs/#config=ONHzqgf2E5Xtmnpsh2gURB;loc=hg19::chr17:55333785.55334472.

### Analysis of Gene ATLAS/UK Biobank GWAS data

We examined SNPs in certain association signals for whether they were also associated with UK Biobank traits that had been analyzed by the Gene ATLAS (http://geneatlas.roslin.ed.ac.uk)^63–65^. For skin-spots signals, we merged imputed GWAS data for two traits (“Ease of skin tanning” and “Skin colour”) from the Gene ATLAS download page with our freckles GWAS summary statistics and examined the overlap between the top freckles or UKBB SNPs. In the case of the excessive hairiness phenotype, we used the Gene ATLAS PheGWAS function to identify associations with other traits in the *TBX15* gene region. The top SNP for multiple anthropometric traits was also our top genotyped *TBX15* variant (rs984222), for which we then extracted beta-coefficients and association P-values for several traits using their Phewas function.

### Figure plotting

Figures that were not associated with outside software/web-services were plotted using self-written R programs. Figures of epigenetic states were produced from RoadMap Epigenomics data using the Washington University Epigenome Browser (http://epigenomegateway.wustl.edu/browser/)^141^ using their “Screenshot” function to produce publication quality images. Genes models shown come from GENCODE V19^142^. BED files of top SNPs in association signals were added as custom tracks. Custom tracks were added for imputed 25-state model from the RoadMap Epigenomics Project as compressed state tracks and as epilogos visualization. The custom track for 25-state model: http://egg2.wustl.edu/roadmap/data/byFileType/chromhmmSegmentations/ChmmModels/compressedStateTracks/hg19_chromHMM_imputed25.gz. Custom track for epilogos visualization: http://egg2.wustl.edu/roadmap/data/byFileType/chromhmmSegmentations/ChmmModels/epilogos/imputed/qcat.gz.

### Data availability

Due to a concern for subject privacy and restrictions in the study consent form, the genotype data for this study is not publicly available to outside researchers. However, we do make the genome-wide summary statistics (β-coefficient and SE, P-value, effect-allele frequency) available as Supplementary Datasets S1–S7; a description and legend for each dataset is available in the Supplementary Information. Reasonable requests for other data should be addressed to Dr. Todd A. Johnson (todd.johnson@stagen.co.jp).

## Electronic supplementary material

Supplementary Information

Supplementary Worksheets S1-S8

Supplementary Worksheets S9-S14

Dataset S1

Dataset S2

Dataset S3

Dataset S4

Dataset S5

Dataset S6

Dataset S7
