# Supplementary material for: Genome-wide association study in Japanese females identifies fifteen novel skin-related trait associations
Source: Sci Rep. 2018 Jun 12;8:8974. doi: 10.1038/s41598-018-27145-2 (PMC5997657; doi:10.1038/s41598-018-27145-2)
Supplement: Supplementary file 1 — Supplementary Information [file 41598_2018_27145_MOESM1_ESM.pdf]

Supplementary Information for:

Genome-wide association study in Japanese females identifies fifteen novel skin-related trait associations

Chihiro Endo[1,¶], Todd A. Johnson[2,¶,*], Ryoko Morino[3], Kazuyuki Nakazono[2], Shigeo Kamitsuji[2], Masanori Akita[3], Maiko Kawajiri[3], Tatsuya Yamasaki[4], Azusa Kami[3], Yuria Hoshi[4], Asami Tada[3], Kenichi Ishikawa[3], Maaya Hine[5], Miki Kobayashi[5], Nami Kurume[5], Yuichiro Tsunemi[1], Naoyuki Kamatani[2], and Makoto Kawashima[1]

[1]Department of Dermatology, School of Medicine, Tokyo Women's Medical University, Shinjuku, Tokyo, 162-8666, Japan

[2]StaGen Co., Ltd., Taito-ku, Tokyo, 111-0051, Japan

[3]EverGene Ltd., Shinjuku-ku, Tokyo, 163-1435, Japan

[4]Life Science Group, Healthcare Division, Department of Healthcare Business, MTI Ltd., Shinjuku-ku, Tokyo, 163-1435, Japan

[5]LunaLuna Division, Department of Healthcare Business, MTI Ltd., Shinjuku-ku, Tokyo, 163-1435, Japan

¶These authors contributed equally to this work.

*Corresponding author

E-mail: todd.johnson@stagen.co.jp (T.A.J.)

Table of Contents

Supplementary Datasets 4

Dataset S1. Age-spots GWAS summary statistics 4

Dataset S2. Freckles GWAS summary statistics 4

Dataset S3. Double eyelid GWAS summary statistics 4

Dataset S4. Eyebrows GWAS summary statistics 4

Dataset S5. Hair (straight/curly) GWAS summary statistics 4

Dataset S6. Excessive hairiness GWAS summary statistics 4

Dataset S7. Excessive sweating GWAS summary statistics 4

Supplementary Tables 5

Table S1. Phenotype and case/control sample information 5

Table S2. Previous freckles GWAS results in current dataset. 6

Table S3. Previous hair density related GWAS results in current dataset. 8

Table S3a: Beard thickness signals in excessive hairiness data 8
Table S3b: Eyebrow thickness in eyebrows (thick/thin) data 8
Table S3c: Eyebrow thickness in excessive hairiness data 8
Table S3d: Unibrow in eyebrows (thick/thin) data 9
Table S3e: Unibrow in excessive hairiness data 10

Table S4. Previous hair morphology related GWAS results in current hair (straight/curly) data. 11

Table S5. Full model of age-spots/freckles associated variants. 11

Supplementary Worksheet legends 12

Supplementary Excel file #1 (Worksheets S1-S8): Association signal summary statistics: 12

Worksheet S1. Association signal summaries 12
Worksheet S2. Top SNPs associated with age spots 12
Worksheet S3. Top SNPs associated with freckles 12
Worksheet S4. Top SNPs associated with double-edged eyelids 12
Worksheet S5. Top SNPs associated with eyebrows (thick vs. thin) 12
Worksheet S6. Top SNPs associated with hair type (straight vs. curly) 12
Worksheet S7. Top SNPs associated with excessive hairiness 12
Worksheet S8. Top SNPs associated with excessive sweating 12

Supplementary Excel file #2 (Worksheets S9-S14): GWAS/eQTL colocalization analyses 12

Worksheet S9. Summary of ABF colocalization analysis of GWAS and multi-tissue eQTL data 12
Worksheet S10. Per-SNP results of ABF colocalization analysis of GWAS and multi-tissue eQTL data 12
Worksheet S11. Summary of ABF colocalization analysis of GWAS and single-tissue eQTL data 12

Worksheet S12. Per-SNP results of ABF colocalization analysis of GWAS and single-tissue eQTL data 12
Worksheet S13. SMR colocalization analysis of GWAS and multi-tissue eQTL data 12
Worksheet S14. SMR colocalization analysis of GWAS and single-tissue eQTL data 13

Supplementary Figures 14

Figure S1. Population structure analysis. 14

Figure S2. QQ plots of genome-wide meta-analysis association statistics. 15

Figure S3. Chr9:16.79-16.81 Mb (BNC2) freckles locus. 16

Figure S4. Chr5:149.19-149.23 Mb (PPARGC1B) skin-spots locus. 17

Figure S7. Chr10:118.45-118.48Mb (HSPA12A) freckles locus haplotype structure. 20

Figure S8. Chr10:118.45-118.48Mb (HSPA12A) freckles locus eQTL analysis. 21

Figure S9. Chr17:55.21-55.25 Mb (AKAP1/MSI2) freckles locus. 22

Figure S10. Genetic risk score (GRS) for freckles positivity. 23

Figure S11. Chr2:108.93-109.57 Mb (EDAR/GCC2/LIMS1) hair morphology and density loci. 24

Figure S12. Chr2:108.93-109.57 Mb (GCC2/LIMS1) excessive hairiness locus eQTL analysis. 25

Figure S13. Chr2:28.82-29.05 Mb (PLB1/PPP1CB) excessive sweating locus eQTL analysis. 26

Figure S14. Chr16:48.26-48.45 Mb (ABCC11/LONP2) excessive sweating locus. 27

Supplementary Datasets

Zipped archives of tabbed text files of GWAS summary statistics for genome-wide genotyped SNPs plus imputed variants in region around association signals.

Common columns:

regional.analysis: Status flag with 0 if SNP data comes from GWAS micro-array data or 1 if SNP lies in imputed region.

CHR: Chromosomes numbered 1-23 for autosomes and chrX.

BP: Basepair position in hg19 coordinates

rsid: dbSNP147 rsID or concatenated ID with chrNN:BP.A2.A1

A1: Effect-allele and non-reference (alternate/ALT) allele in hg19

A2: hg19 reference/REF allele

A1.AF: Mean frequency of A1 allele across LL01 and LL02 study stages

P: Unconditioned P-value from linear (bust-size and dysmenorrhea) or logistic (menstrual fever) regression

BETA: Unconditioned beta-coefficient from linear (bust-size and dysmenorrhea) or logistic (menstrual fever) regression

SE: Unconditioned beta-coefficient standard error (SE) from linear (bust-size and dysmenorrhea) or logistic (menstrual fever) regression

Dataset S1. Age-spots GWAS summary statistics

Dataset S2. Freckles GWAS summary statistics

Dataset S3. Double eyelid GWAS summary statistics

Dataset S4. Eyebrows GWAS summary statistics

Dataset S5. Hair (straight/curly) GWAS summary statistics

Dataset S6. Excessive hairiness GWAS summary statistics

Dataset S7. Excessive sweating GWAS summary statistics

Supplementary Tables

Table S1. Phenotype and case/control sample information

Age is presented as mean (SD).

Phenotype	Covariates	LL01 sample counts (Total=5697)			LL02 sample counts (Total=5614)			Age	
		Missing	Controls	Cases	Missing	Controls	Cases	Controls	Cases
**Single-stage analyses**									
Double-edged eyelids	PC1,PC2,Age	0	0	0	0	2069	3545	31.7(6.6)	32.66(6.89)
Eyebrows (thick/thin)	PC1,PC2,Age	0	0	0	1673	1153	2788	32.47(6.57)	31.78(6.76)
Hair (straight/curly)	PC1,PC2,Age,BMI	0	0	0	736	2532	2346	32.7(6.84)	31.84(6.69)
Excessive sweating	PC1,PC2,BMI	0	0	0	1076	1245	3293	32.39(6.71)	32.01(6.77)
**Discovery/evaluation analyses**									
Age spots	PC1,PC2,Age	58	1861	3778	0	1954	3660	30.13(6.37)	30.13(6.37)
Freckles	PC1,PC2,Age	129	3563	2005	0	3585	2029	32.13(6.68)	33.16(6.66)
Excessive hairiness	PC1,PC2,Age,BMI	67	1962	3668	0	1868	3746	33.44(6.75)	32.03(6.6)

Table S2. Previous freckles GWAS results in current dataset.

We extracted previously reported freckles SNPs from Supplementary Table 1 of Jacobs et al and Table 2 of Eriksson et al, merged with 1000 Genomes Project site information (EAS and EUR allele frequencies), and with the current study's summary statistics based genome-wide imputed freckles data. Rows highlighted in grey are SNPs with *FDR*<0.1.

			1000G EA		Previous report					Current study				
rsid	Chr	Position	EAS	EUR	Effect Allele	Author	Gene	P	Beta	Imp. Info	z	P	FDR	SNP status
rs12203592	6	396321	0.00	0.12	T	Eriksson	*IRF4*	2.1E-91	1.61					Mono.
rs872071	6	411064	0.31	0.47	G	Eriksson	*IRF4*	4.2E-15	0.51	1.00	1.64	0.101	0.322	
rs3778607	6	403799	0.26	0.49	A	Eriksson	*IRF4*	2.1E-13	-0.47	1.00	1.63	0.104	0.322	
rs9328192	6	434364	0.36	0.46	G	Eriksson	*IRF4*	4.1E-10	0.40	1.00	2.44	0.015	0.097	
rs9405675	6	444600	0.36	0.63	G	Eriksson	*IRF4*	1.9E-09	-0.39	0.93	2.53	0.011	0.091	
rs12203592	6	396321	0.00	0.12	T	Jacobs	*IRF4*	1.8E-27	0.44					Mono.
rs62389423	6	421281	0.00	0.09	A	Jacobs	*IRF4*	5.5E-20	0.46					Mono.
rs62389424	6	422631	0.12	0.10	A	Jacobs	*IRF4*	2.8E-15	0.41	0.83	2.35	0.019	0.111	
rs2153271	9	16864521	0.23	0.58	C	Eriksson	*BNC2*	4.0E-10	-0.40	1.00	2.85	4.4E-03	0.091	
rs62543565	9	16901067	0.34	0.62	C	Jacobs	*BNC2*	1.5E-07	-0.15	0.94	2.11	0.035	0.169	
rs12931267	16	89818732	0.02	0.07	G	Eriksson	*MC1R*	8.3E-62	1.88	0.46	-1.82	0.069	0.304	Low freq.
rs8049897	16	90024202	0.27	0.13	A	Eriksson	*MC1R*	1.6E-30	1.03	0.81	2.80	5.1E-03	0.091	
rs1805008	16	89986144	0.00	0.06	T	Eriksson	*MC1R*	6.6E-24	1.21					Mono.
rs1805009	16	89986546	0.00	0.01	C	Eriksson	*MC1R*	4.8E-18	2.02					Mono.
rs11547464	16	89986091	0.00	0.01	A	Eriksson	*MC1R*	1.4E-13	2.43					Mono.
rs11861084	16	89875710	1.00	0.60	A	Eriksson	*MC1R*	5.4E-12	-0.45					Mono.
rs7204478	16	89795485	0.73	0.48	T	Eriksson	*MC1R*	2.5E-11	0.43	0.87	-2.19	0.028	0.151	
rs7195066	16	89836323	0.99	0.32	T	Eriksson	*MC1R*	7.6E-11	-0.45					Low freq.
rs11648785	16	90084561	0.16	0.34	T	Eriksson	*MC1R*	1.1E-09	-0.42	0.80	-1.18	0.240	0.488	
rs8060934	16	89920025	0.73	0.55	C	Eriksson	*MC1R*	1.4E-09	-0.39	0.81	-3.00	2.7E-03	0.091	
rs154659	16	89667337	0.39	0.25	C	Eriksson	*MC1R*	2.4E-09	0.43	0.49	-1.71	0.088	0.322	Low info.
rs35850949	16	89720724	0.00	0.07	A	Jacobs	*MC1R*	8.7E-15	0.33					Mono.
rs71396950	16	89726550	0.00	0.07	A	Jacobs	*MC1R*	8.7E-15	0.35					Mono.
rs71396951	16	89726593	0.00	0.07	C	Jacobs	*MC1R*	8.7E-15	0.33					Mono.
rs35063026	16	89736157	0.00	0.07	T	Jacobs	*MC1R*	8.7E-15	0.33					Mono.
rs35749174	16	89716493	0.00	0.07	A	Jacobs	*MC1R*	1.4E-14	0.35					Mono.
rs71396949	16	89714844	0.00	0.07	A	Jacobs	*MC1R*	3.3E-14	0.37					Mono.
rs112460025	16	90051337	0.01	0.07	G	Jacobs	*MC1R*	1.9E-13	0.35	0.24	0.89	0.374	0.488	Mono.
rs74800773	16	90024970	0.00	0.08	G	Jacobs	*MC1R*	2.9E-13	0.35	0.21	0.18	0.857	0.890	Mono.
rs146972365	16	90022693	0.00	0.07	C	Jacobs	*MC1R*	4.6E-13	0.36	0.31	0.58	0.563	0.620	Mono.
rs112233725	16	89693191	0.06	0.07	C	Jacobs	*MC1R*	7.6E-13	0.37	0.40	0.07	0.947	0.947	Low info.
rs77606435	16	90053691	0.00	0.07	G	Jacobs	*MC1R*	7.9E-13	0.35	0.26	0.95	0.345	0.488	Mono.
rs73283859	16	90062520	0.01	0.07	C	Jacobs	*MC1R*	7.9E-13	0.35	0.30	0.99	0.321	0.488	Mono.
rs73283869	16	90067184	0.01	0.07	A	Jacobs	*MC1R*	7.9E-13	0.35	0.30	1.01	0.312	0.488	Mono.
rs77381714	16	90078022	0.00	0.07	C	Jacobs	*MC1R*	8.1E-13	0.41					Mono.
rs112119225	16	90063461	0.01	0.07	G	Jacobs	*MC1R*	1.0E-12	0.36	0.27	0.91	0.365	0.488	Mono.
rs75319471	16	90064454	0.01	0.07	G	Jacobs	*MC1R*	1.0E-12	0.36	0.27	0.95	0.340	0.488	Mono.
rs74336735	16	90065033	0.01	0.07	A	Jacobs	*MC1R*	1.0E-12	0.36	0.27	0.83	0.404	0.488	Mono.
rs76581091	16	90065100	0.01	0.07	C	Jacobs	*MC1R*	1.0E-12	0.36	0.27	0.83	0.404	0.488	Mono.
rs59038611	16	90065956	0.01	0.07	C	Jacobs	*MC1R*	1.0E-12	0.37	0.28	0.85	0.393	0.488	Mono.
rs73283867	16	90066260	0.01	0.07	G	Jacobs	*MC1R*	1.0E-12	0.37	0.26	0.94	0.350	0.488	Mono.
rs78800020	16	90067136	0.01	0.07	C	Jacobs	*MC1R*	1.0E-12	0.37	0.26	0.91	0.363	0.488	Mono.
rs73283871	16	90067202	0.01	0.07	C	Jacobs	*MC1R*	1.0E-12	0.37	0.26	0.91	0.363	0.488	Mono.
rs59574756	16	90067513	0.01	0.07	G	Jacobs	*MC1R*	1.0E-12	0.37	0.26	0.95	0.345	0.488	Mono.
rs45610233	16	90048395	0.00	0.07	T	Jacobs	*MC1R*	3.2E-12	0.35					Mono.
rs113891247	16	90047757	0.01	0.07	A	Jacobs	*MC1R*	3.8E-12	0.35	0.31	1.03	0.302	0.488	Mono.
rs113955373	16	90043036	0.01	0.07	A	Jacobs	*MC1R*	7.1E-12	0.35	0.26	0.89	0.376	0.488	Mono.
rs77770855	16	90043010	0.01	0.07	A	Jacobs	*MC1R*	8.4E-12	0.35	0.27	0.85	0.395	0.488	Mono.
rs56850194	16	90043531	0.01	0.07	A	Jacobs	*MC1R*	8.4E-12	0.35	0.27	0.84	0.399	0.488	Mono.
rs58827852	16	90058754	0.01	0.08	A	Jacobs	*MC1R*	1.2E-11	0.35	0.28	0.83	0.408	0.488	Mono.
rs112556696	16	90054018	0.01	0.10	G	Jacobs	*MC1R*	2.6E-11	0.65	0.24	0.75	0.451	0.520	Low freq.
rs75923656	16	89690990	0.20	0.07	C	Jacobs	*MC1R*	3.6E-11	0.36	0.45	-1.18	0.238	0.488	Low info.
rs79138604	16	89690991	0.20	0.07	A	Jacobs	*MC1R*	3.6E-11	0.36	0.47	-1.26	0.209	0.481	Low info.
rs3764253	16	89686693	0.20	0.07	A	Jacobs	*MC1R*	5.2E-11	0.36	0.49	-1.57	0.115	0.322	Low info.

rs3764253	16	89686693	0.00	0.00	A	Jacobs	*MC1R*	5.2E-11	0.36	0.49	-1.57	0.115	0.322	Mono.
rs2280374	16	89687407	0.21	0.07	T	Jacobs	*MC1R*	5.2E-11	0.36	0.48	-1.51	0.131	0.332	Low info.
rs79172130	16	89687812	0.21	0.07	A	Jacobs	*MC1R*	5.2E-11	0.36	0.48	-1.51	0.131	0.332	Low info.
rs4968054	16	89690079	0.20	0.07	T	Jacobs	*MC1R*	5.2E-11	0.36	0.51	-1.62	0.105	0.322	Low info.
rs34323930	16	89691045	0.20	0.07	T	Jacobs	*MC1R*	6.5E-11	0.33	0.46	-1.26	0.207	0.481	Low info.
rs77110324	16	89683288	0.00	0.06	A	Jacobs	*MC1R*	7.5E-10	0.42	0.35	0.56	0.573	0.620	Mono.
rs8063761	16	90027626	0.28	0.28	T	Jacobs	*MC1R*	8.1E-10	0.20	0.79	2.60	9.4E-03	0.091	
rs62052243	16	90026152	0.28	0.28	G	Jacobs	*MC1R*	1.4E-09	0.20	0.79	2.53	0.011	0.091	
rs8051733	16	90024206	0.28	0.28	G	Jacobs	*MC1R*	3.1E-09	0.21	0.79	2.51	0.012	0.091	
rs34395984	16	89659511	0.00	0.06	T	Jacobs	*MC1R*	3.8E-09	0.37					Mono.
rs7195043	16	90020861	0.83	0.44	T	Jacobs	*MC1R*	2.9E-08	0.23	0.77	1.64	0.101	0.322	
rs619865	20	33867697	1.00	0.93	A	Eriksson	*ASIP*	5.5E-14	0.77					Mono.
rs291671	20	31950845	0.69	0.93	G	Eriksson	*ASIP*	9.1E-13	0.77	0.93	0.07	0.945	0.947	
rs6088316	20	32426842	0.02	0.13	G	Eriksson	*ASIP*	4.0E-11	0.57	0.18	-0.24	0.810	0.859	Low freq.
rs4911442	20	33355046	1.00	0.91	G	Eriksson	*ASIP*	5.8E-11	0.63					Mono.
rs761238	20	32519988	0.85	0.69	G	Eriksson	*ASIP*	1.3E-10	0.43	0.96	0.82	0.411	0.488	
rs17305657	20	31806588	0.00	0.06	C	Eriksson	*ASIP*	1.5E-10	0.70					Mono.
rs4812405	20	35276585	0.00	0.04	A	Eriksson	*ASIP*	2.0E-10	0.78					Mono.
rs1474976	20	34732372	1.00	0.93	C	Eriksson	*ASIP*	3.2E-10	0.62					Mono.
rs4911414	20	32729444	0.83	0.70	T	Eriksson	*ASIP*	3.0E-09	0.39	1.00	0.82	0.414	0.488	
rs6059655	20	32665748	1.00	0.96	A	Jacobs	*ASIP*	1.9E-09	0.30					Mono.
rs62209647	20	32505658	0.00	0.04	C	Jacobs	*ASIP*	2.6E-09	0.33					Mono.
rs62211989	20	32538391	0.00	0.04	C	Jacobs	*ASIP*	3.0E-09	0.34					Mono.
rs6142422	20	34216058	1.00	0.96	C	Jacobs	*ASIP*	1.1E-08	0.26	0.18	0.63	0.528	0.595	Mono.
rs56069863					T	Jacobs		5.8E-13	0.48					Not in 1000G
rs140889249					G	Jacobs		1.9E-12	0.41					Not in 1000G

Table S3. Previous hair density related GWAS results in current dataset.

We extracted previously reported SNPs from Strongest Associations table for unibrow phenotype from 2016 Pickrell et al, and hair related data from Table 1 and Supplementary Table 6 of 2016 Adhikari et al, merged with 1000 Genomes Project site information (EAS and EUR allele frequencies), and with the current study's summary statistics based genome-wide imputed excessive hairiness or eyebrow thickness data. Note that Adhikari, et al. did not report the effect allele/size. Rows highlighted in grey are SNPs with *FDR*<0.1.

Table S3a: Beard thickness signals in excessive hairiness data

			1000G EA		Previous report		Current study				
rsid	Chr	Position	EAS	EUR	Author	P	Imp. Info	z	P	FDR	SNP status
rs11121667	1	11038476	0.29	0.24	Adhikari	3.8E-07	1.00	-4.68	2.9E-06	2.0E-05	
rs6684877	1	39557810	0.00	0.09	Adhikari	8.2E-06					Mono.
rs365060	2	109575736	0.95	0.09	Adhikari	1.0E-15	0.51	-1.94	0.053	0.185	Low info.
rs4864809	4	54511913	0.59	0.36	Adhikari	1.0E-08	0.97	0.05	0.957	0.987	
rs1003136	5	127347815	0.43	0.33	Adhikari	1.1E-06	1.00	-0.02	0.987	0.987	
rs6901317	6	106036151	0.32	0.69	Adhikari	4.0E-08	1.00	0.53	0.596	0.838	
rs117717824	7	113989202	0.05	0.00	Adhikari	1.0E-08	0.65	-0.53	0.598	0.838	Low freq.
rs10788096	10	122242293	0.69	0.42	Adhikari	1.5E-06	0.94	1.00	0.319	0.744	

Table S3b: Eyebrow thickness in eyebrows (thick/thin) data

			1000G EA		Previous report		Current study				
rsid	Chr	Position	EAS	EUR	Author	P	Imp. Info	z	P	FDR	SNP status
rs10908366	1	38121934	0.42	0.50	Adhikari	6.5E-06	0.85	-0.04	0.972	0.999	
rs3827760	2	109513601	0.87	0.01	Adhikari	1.2E-07	1.00	6.02	1.7E-09	3.4E-08	
rs112458845	3	138675741	0.03	0.00	Adhikari	4.0E-10	0.71	0.07	0.946	0.999	Low freq.
rs1868245	4	87848739	0.83	1.00	Adhikari	2.3E-06	0.91	0.18	0.856	0.999	
rs9654415	5	72544390	0.13	0.39	Adhikari	2.6E-07	0.94	0.84	0.399	0.999	
rs10515457	5	132650871	0.20	0.16	Adhikari	2.8E-06	0.85	-0.42	0.675	0.999	
rs201287033	7	150823227	0.24	0.00	Adhikari	7.2E-06	0.92	-0.99	0.321	0.999	
rs62578126	9	129375338	0.13	0.37	Adhikari	3.4E-06	0.65	0.00	0.999	0.999	
rs112386516	15	78375229	0.12	0.05	Adhikari	1.5E-06	0.88	0.65	0.517	0.999	
rs12597422	16	53887738	0.64	0.21	Adhikari	3.0E-06	0.96	-0.64	0.521	0.999	

Table S3c: Eyebrow thickness in excessive hairiness data

			1000G EA		Previous report		Current study				
rsid	Chr	Position	EAS	EUR	Author	P	Imp. Info	z	P	FDR	SNP status
rs10908366	1	38121934	0.42	0.50	Adhikari	6.5E-06	0.85	0.70	0.485	0.999	
rs3827760	2	109513601	0.87	0.01	Adhikari	1.2E-07	1.00	-5.84	5.1E-09	5.1E-08	
rs112458845	3	138675741	0.03	0.00	Adhikari	4.0E-10	0.70	-1.51	0.130	0.650	Low freq.
rs1868245	4	87848739	0.83	1.00	Adhikari	2.3E-06	0.91	0.30	0.762	0.999	
rs9654415	5	72544390	0.13	0.39	Adhikari	2.6E-07	0.94	1.92	0.055	0.364	
rs10515457	5	132650871	0.20	0.16	Adhikari	2.8E-06	0.85	-0.26	0.795	0.999	
rs201287033	7	150823227	0.24	0.00	Adhikari	7.2E-06	0.92	0.13	0.899	0.999	
rs62578126	9	129375338	0.13	0.37	Adhikari	3.4E-06	0.64	0.26	0.792	0.999	
rs112386516	15	78375229	0.12	0.05	Adhikari	1.5E-06	0.88	-0.95	0.342	0.999	
rs12597422	16	53887738	0.64	0.21	Adhikari	3.0E-06	0.96	0.22	0.827	0.999	

Table S3d: Unibrow in eyebrows (thick/thin) data

			1000G EA		Previous report				Current study				
rsid	Chr	Position	EAS	EUR	Effect Allele	Author	P	Beta	Imp. Info	z	P	FDR	SNP status
rs702490	1	54195018	0.52	0.68	G	Pickrell	2.4E-45	-0.06	1.00	0.40	0.687	0.874	
rs7542091	1	210031948	0.64	0.38	G	Pickrell	1.5E-11	-0.03	0.96	1.07	0.285	0.703	
rs6676180	1	119783223	0.43	0.38	T	Pickrell	4.3E-11	0.03	0.90	1.10	0.272	0.695	
rs1267303	1	46990457	0.54	0.73	G	Pickrell	3.6E-09	0.03	0.85	0.06	0.955	0.995	
rs2218065	2	223034082	0.40	0.64		Adhikari	5.0E-08		0.96	0.29	0.770	0.933	
rs3827760	2	109513601	0.87	0.01		Adhikari	1.5E-07		1.00	6.02	1.7E-09	7.8E-08	
rs2053316	2	223094489	0.02	0.07	T	Pickrell	7.9E-104	0.15	0.68	0.43	0.664	0.874	Low freq.
rs12467812	2	19995470	0.47	0.33	G	Pickrell	3.3E-17	0.03	0.86	1.20	0.231	0.685	
rs3827760	2	109513601	0.87	0.01	G	Pickrell	2.4E-15	0.17	1.00	6.02	1.7E-09	7.8E-08	
rs970797	2	177111819	0.69	0.57	T	Pickrell	9.0E-12	-0.03	0.96	-0.41	0.685	0.874	
rs11694441	2	119541381	0.00	0.13	C	Pickrell	8.4E-10	0.04					Mono.
rs1345417	3	181511951	0.29	0.63	G	Pickrell	1.7E-31	0.05	0.89	4.43	9.5E-06	1.2E-04	
rs76036841	3	147804236	0.00	0.06	G	Pickrell	1.5E-09	0.06					Mono.
rs724818	4	121627113	0.11	0.04		Adhikari	4.2E-06		0.95	-0.82	0.413	0.730	
rs6828882	4	3166196	0.01	0.10	G	Pickrell	1.3E-22	-0.06	0.21	0.70	0.482	0.775	Mono.
rs3796606	4	81194090	0.77	0.40	G	Pickrell	5.50E-10	0.02	0.95	1.15	0.250	0.695	
rs7702331	5	72551134	0.13	0.39		Adhikari	4.30E-07		0.94	0.83	0.407	0.730	
rs16891982	5	33951693	0.01	0.94	G	Pickrell	6.80E-48	-0.15	0.40	1.91	0.056	0.250	Mono.
rs6881396	5	72559339	0.14	0.41	T	Pickrell	6.70E-35	-0.05	0.93	0.77	0.444	0.742	
rs199955185	5	153938270	0.38	0.64	I	Pickrell	1.80E-20	-0.04	0.91	-0.67	0.505	0.787	
rs1553477	5	53116966	0.22	0.50	C	Pickrell	2.30E-17	-0.03	0.96	-1.35	0.178	0.585	
rs4476718	5	124075470	0.87	0.81	T	Pickrell	2.20E-14	-0.04	1.00	-2.66	7.9E-03	0.066	
rs12197419	6	1709037	0.55	0.75		Adhikari	1.70E-06		1.00	1.50	0.133	0.509	
rs9449896	6	85118988	0.05	0.17	C	Pickrell	1.90E-19	-0.04	0.73	2.43	0.015	0.107	
rs227833	6	44681840	0.27	0.28	G	Pickrell	5.60E-16	0.03	0.94	2.32	0.020	0.134	
rs61123157	6	106613530	0.49	0.39	T	Pickrell	4.60E-14	0.03	0.93	0.18	0.854	0.982	
rs7763441	6	2452697	0.76	0.43	T	Pickrell	4.30E-13	0.03	0.96	0.05	0.963	0.995	
rs12203592	6	396321	0.00	0.12	T	Pickrell	1.40E-10	0.03					Mono.
rs1178101	7	18737197	0.30	0.17	C	Pickrell	7.30E-16	0.04	0.96	-0.03	0.980	0.995	
rs72328411	7	40319239	0.01	0.02	I	Pickrell	2.90E-10	-0.08					Low freq.
rs3094901	7	69937950	0.33	0.51	T	Pickrell	2.20E-09	-0.02	1.00	-0.38	0.703	0.874	
rs7837649	8	77658645	0.43	0.17	G	Pickrell	6.00E-21	-0.05	0.97	0.01	0.992	0.995	
rs12236890	9	3157137	0.66	0.43		Adhikari	2.00E-06		1.00	-0.40	0.688	0.874	
rs7861975	9	1121353	0.70	0.40	T	Pickrell	4.20E-62	0.06	0.96	-0.69	0.488	0.775	
rs3829849	9	129390800	0.13	0.37	T	Pickrell	1.20E-15	0.03	0.64	0.09	0.930	0.995	
rs111677654	9	98877903	0.15	0.16	T	Pickrell	3.80E-11	-0.03	0.93	-1.69	0.091	0.363	
rs7905367	10	54334653	0.06	0.77	G	Pickrell	4.40E-15	-0.04	1.00	3.43	6.0E-04	6.1E-03	
rs113334738	10	18271858	0.02	0.15	G	Pickrell	4.30E-09	0.03	0.61	-1.14	0.255	0.695	Low freq.
rs66716358	11	44330610	0.51	0.47	T	Pickrell	7.60E-13	-0.03	0.93	-3.59	3.3E-04	3.8E-03	
rs10626466	11	69683380	0.57	0.42	I	Pickrell	4.60E-12	0.03	0.94	-0.99	0.322	0.703	
rs10500859	11	19740731	0.02	0.13	G	Pickrell	1.50E-11	-0.04	0.89	0.02	0.981	0.995	Low freq.
rs138341189	11	68224411	0.20	0.15	I	Pickrell	1.90E-09	0.04	0.84	0.40	0.687	0.874	
rs4760786	12	71446789	0.01	0.32	T	Pickrell	8.70E-21	0.04	0.63	-0.81	0.420	0.730	Low freq.
rs11609649	12	85576492	0.00	0.16	G	Pickrell	1.30E-12	0.03	0.21	-0.22	0.827	0.975	Mono.
rs11105799	12	91153721	0.03	0.30	T	Pickrell	1.80E-12	0.03	0.41	0.03	0.979	0.995	Low freq.
rs4144266	14	92789205	0.46	0.43	G	Pickrell	4.20E-21	-0.04	0.90	-0.84	0.399	0.730	
rs12916300	15	28410491	0.00	0.66	T	Pickrell	4.60E-47	-0.07	0.19	-0.03	0.974	0.995	Mono.
rs3743103	15	33025627	0.58	0.59	T	Pickrell	5.30E-25	-0.04	0.94	1.00	0.315	0.703	
rs78498439	16	89967514	0.00	0.05	G	Pickrell	6.90E-14	0.08					Mono.
rs536204166	17	43849572	0.00	0.24	I	Pickrell	2.80E-32	-0.05					Mono.
rs230203	20	55791615	0.33	0.58	T	Pickrell	1.70E-19	-0.03	0.94	2.10	0.036	0.195	
rs6082544	20	21868713	0.59	0.58	G	Pickrell	3.50E-09	-0.02	0.89	1.33	0.184	0.585	
rs2804362	23	69001854	0.01	0.57	G	Pickrell	8.90E-36	-0.04					Mono.
rs1187437						Adhikari	3.50E-07						Not in 1000G
rs72422730					I	Pickrell	6.40E-21	-0.04					Not in 1000G
rs67922471					I	Pickrell	1.40E-15	-0.03					Not in 1000G
rs199883506					I	Pickrell	1.30E-13	-0.04					Not in 1000G

Table S3e: Unibrow in excessive hairiness data

			1000G EA		Previous report				Current study				
rsid	Chr	Position	EAS	EUR	Effect Allele	Author	P	Beta	Imp. Info	z	P	FDR	SNP status
rs702490	1	54195018	0.52	0.68	G	Pickrell	2.4E-45	-0.06	1.00	0.08	0.937	0.995	
rs7542091	1	210031948	0.64	0.38	G	Pickrell	1.5E-11	-0.03	0.96	0.22	0.823	0.975	
rs6676180	1	119783223	0.43	0.38	T	Pickrell	4.3E-11	0.03	0.90	-1.46	0.143	0.525	
rs1267303	1	46990457	0.54	0.73	G	Pickrell	3.6E-09	0.03	0.85	2.07	0.038	0.195	
rs2218065	2	223034082	0.40	0.64		Adhikari	5.0E-08		0.95	0.45	0.655	0.874	
rs3827760	2	109513601	0.87	0.01		Adhikari	1.5E-07		1.00	-5.84	5.1E-09	1.2E-07	
rs2053316	2	223094489	0.02	0.07	T	Pickrell	7.9E-104	0.15	0.68	0.99	0.322	0.703	Low freq.
rs12467812	2	19995470	0.47	0.33	G	Pickrell	3.3E-17	0.03	0.86	0.55	0.585	0.855	
rs3827760	2	109513601	0.87	0.01	G	Pickrell	2.4E-15	0.17	1.00	-5.84	5.1E-09	1.2E-07	
rs970797	2	177111819	0.69	0.57	T	Pickrell	9.0E-12	-0.03	0.96	-0.38	0.703	0.874	
rs11694441	2	119541381	0.00	0.13	C	Pickrell	8.4E-10	0.04				NA	Mono.
rs1345417	3	181511951	0.29	0.63	G	Pickrell	1.7E-31	0.05	0.89	5.78	7.6E-09	1.4E-07	
rs76036841	3	147804236	0.00	0.06	G	Pickrell	1.5E-09	0.06				NA	Mono.
rs724818	4	121627113	0.11	0.04		Adhikari	4.2E-06		0.95	0.29	0.769	0.933	
rs6828882	4	3166196	0.01	0.10	G	Pickrell	1.3E-22	-0.06	0.21	0.17	0.866	0.982	Mono.
rs3796606	4	81194090	0.77	0.40	G	Pickrell	5.50E-10	0.02	0.95	-0.61	0.541	0.821	
rs7702331	5	72551134	0.13	0.39		Adhikari	4.30E-07		0.94	1.90	0.057	0.250	
rs16891982	5	33951693	0.01	0.94	G	Pickrell	6.80E-48	-0.15	0.40	-0.74	0.461	0.758	Mono.
rs6881396	5	72559339	0.14	0.41	T	Pickrell	6.70E-35	-0.05	0.93	2.04	0.042	0.202	
rs199955185	5	153938270	0.38	0.64	I	Pickrell	1.80E-20	-0.04	0.91	-0.98	0.327	0.703	
rs1553477	5	53116966	0.22	0.50	C	Pickrell	2.30E-17	-0.03	0.96	-0.80	0.425	0.730	
rs4476718	5	124075470	0.87	0.81	T	Pickrell	2.20E-14	-0.04	1.00	1.78	0.076	0.317	
rs12197419	6	1709037	0.55	0.75		Adhikari	1.70E-06		1.00	-1.45	0.148	0.525	
rs9449896	6	85118988	0.05	0.17	C	Pickrell	1.90E-19	-0.04	0.74	0.79	0.428	0.730	
rs227833	6	44681840	0.27	0.28	G	Pickrell	5.60E-16	0.03	0.94	1.12	0.265	0.695	
rs61123157	6	106613530	0.49	0.39	T	Pickrell	4.60E-14	0.03	0.93	0.88	0.381	0.730	
rs7763441	6	2452697	0.76	0.43	T	Pickrell	4.30E-13	0.03	0.96	-0.88	0.381	0.730	
rs12203592	6	396321	0.00	0.12	T	Pickrell	1.40E-10	0.03				NA	Mono.
rs1178101	7	18737197	0.30	0.17	C	Pickrell	7.30E-16	0.04	0.96	0.98	0.328	0.703	
rs72328411	7	40319239	0.01	0.02	I	Pickrell	2.90E-10	-0.08				NA	Low freq.
rs3094901	7	69937950	0.33	0.51	T	Pickrell	2.20E-09	-0.02	1.00	-0.82	0.411	0.730	
rs7837649	8	77658645	0.43	0.17	G	Pickrell	6.00E-21	-0.05	0.97	0.01	0.995	0.995	
rs12236890	9	3157137	0.66	0.43		Adhikari	2.00E-06		1.00	-0.46	0.646	0.874	
rs7861975	9	1121353	0.70	0.40	T	Pickrell	4.20E-62	0.06	0.96	-0.98	0.326	0.703	
rs3829849	9	129390800	0.13	0.37	T	Pickrell	1.20E-15	0.03	0.63	0.18	0.854	0.982	
rs111677654	9	98877903	0.15	0.16	T	Pickrell	3.80E-11	-0.03	0.92	0.88	0.380	0.730	
rs7905367	10	54334653	0.06	0.77	G	Pickrell	4.40E-15	-0.04	1.00	2.77	5.6E-03	0.051	
rs113334738	10	18271858	0.02	0.15	G	Pickrell	4.30E-09	0.03	0.62	-2.61	0.009	0.070	Low freq.
rs66716358	11	44330610	0.51	0.47	T	Pickrell	7.60E-13	-0.03	0.93	-4.53	5.9E-06	9.1E-05	
rs10626466	11	69683380	0.57	0.42	I	Pickrell	4.60E-12	0.03	0.94	-1.35	0.177	0.585	
rs10500859	11	19740731	0.02	0.13	G	Pickrell	1.50E-11	-0.04	0.89	-0.49	0.627	0.874	Low freq.
rs138341189	11	68224411	0.20	0.15	I	Pickrell	1.90E-09	0.04	0.84	-2.20	0.028	0.159	
rs4760786	12	71446789	0.01	0.32	T	Pickrell	8.70E-21	0.04	0.63	-0.45	0.654	0.874	Low freq.
rs11609649	12	85576492	0.00	0.16	G	Pickrell	1.30E-12	0.03	0.21	0.80	0.426	0.730	Mono.
rs11105799	12	91153721	0.03	0.30	T	Pickrell	1.80E-12	0.03	0.41	1.22	0.224	0.685	Low freq.
rs4144266	14	92789205	0.46	0.43	G	Pickrell	4.20E-21	-0.04	0.90	-0.59	0.553	0.821	
rs12916300	15	28410491	0.00	0.66	T	Pickrell	4.60E-47	-0.07	0.20	0.16	0.875	0.982	Mono.
rs3743103	15	33025627	0.58	0.59	T	Pickrell	5.30E-25	-0.04	0.94	-1.12	0.264	0.695	
rs78498439	16	89967514	0.00	0.05	G	Pickrell	6.90E-14	0.08				NA	Mono.
rs536204166	17	43849572	0.00	0.24	I	Pickrell	2.80E-32	-0.05				NA	Mono.
rs230203	20	55791615	0.33	0.58	T	Pickrell	1.70E-19	-0.03	0.94	2.28	0.022	0.137	
rs6082544	20	21868713	0.59	0.58	G	Pickrell	3.50E-09	-0.02	0.89	0.60	0.546	0.821	
rs2804362	23	69001854	0.01	0.57	G	Pickrell	8.90E-36	-0.04				NA	Mono.
rs1187437						Adhikari	3.50E-07					NA	Not in 1000G
rs72422730					I	Pickrell	6.40E-21	-0.04				NA	Not in 1000G
rs67922471					I	Pickrell	1.40E-15	-0.03				NA	Not in 1000G
rs199883506					I	Pickrell	1.30E-13	-0.04				NA	Not in 1000G

Table S4. Previous hair morphology related GWAS results in current hair (straight/curly) data.

We extracted previously reported SNPs from Strongest Associations table for hair related data from Table 1 and Supplementary Table 6 of 2016 Adhikari et al, merged with 1000 Genomes Project site information (EAS and EUR allele frequencies), and with the current study's summary statistics based genome-wide imputed hair morphology data.

			1000G EA		Previous report		Current study				
rsid	Chr	Position	EAS	EUR	Author	P	Imp. Info	z	P	FDR	SNP status
rs11803731	1	152083325	0.00	0.23	Adhikari	3.00E-12	0.22	0.96	0.338	0.608	Mono.
rs3827760	2	109513601	0.87	0.01	Adhikari	3.00E-119	1.00	21.58	3.10E-103	2.80E-102	
rs4864363	4	137691750	0.54	0.16	Adhikari	4.40E-07	0.98	-1.74	0.081	0.289	
rs73199888	7	109757018	0.20	0.16	Adhikari	1.50E-07	0.91	-0.31	0.756	0.973	
rs17143387	10	8314044	0.47	0.16	Adhikari	4.00E-08	1.00	1.49	0.137	0.308	
rs4881147	10	3342774	0.42	0.71	Adhikari	3.00E-06	1.00	0.66	0.509	0.764	
rs3742377	14	100166228	0.15	0.14	Adhikari	1.40E-07	1.00	0.02	0.982	0.982	
rs11150606	16	31099011	0.76	0.03	Adhikari	7.00E-09	0.47	1.66	0.096	0.289	Low info.
rs4800451	18	20716805	0.81	0.72	Adhikari	5.60E-06	0.92	-0.10	0.924	0.982	

Table S5. Full model of age-spots/freckles associated variants.

Model variants: "Top SNPs" includes top SNP from each association signal; "With HAPS" includes SNPs from haplotype clusters in place of some top SNPs.

Phenotype samples tested	Model variants	Anova P	Pseudo R2	SNPs tested
Freckles	Freckles top SNPs	4.60×10^{-89}	0.0520	rs10810635, rs251468, rs10444039, rs12259842, rs10886142, rs4752116, rs17833789
Freckles (no age-spots controls)	Freckles top SNPs	5.83×10^{-83}	0.0723	rs10810635, rs251468, rs10444039, rs12259842, rs10886142, rs4752116, rs17833789
Age-spots	Age-spots top SNPs	3.16×10^{-23}	0.0136	rs251468, rs61866017, rs35563099
Age-spots (no freckles cases)	Age-spots top SNPs	1.23×10^{-7}	0.0067	rs251468, rs61866017, rs35563099

Table S6. Tabulation of the overlap between freckles and age-spot phenotypes.

	Freckles			
Age-spots	Unknown	Very applicable	Slightly true	Not applicable
**Unknown**	37	7	13	1
**Very applicable**	40	1040	501	637
**Slightly true**	48	447	1565	3160
**Not applicable**	4	96	365	3350

Supplementary Worksheet legends

Supplementary Excel file #1 (Worksheets S1-S8): Association signal summary statistics:

First two worksheets in file contain Legends with descriptions of individual columns in the following worksheets.

Worksheet S1. Association signal summaries
Descriptive information, aggregated statistics, and annotations of association signals. See Supplementary Worksheet 1 legend worksheet for descriptions of individual columns.

Worksheet S2. Top SNPs associated with age spots
Descriptive information, statistics, and annotation of top age spot associated SNPs

Worksheet S3. Top SNPs associated with freckles
Descriptive information, statistics, and annotation of freckle associated SNPs

Worksheet S4. Top SNPs associated with double-edged eyelids
Descriptive information, statistics, and annotation of top double-edged eyelids associated SNPs

Worksheet S5. Top SNPs associated with eyebrows (thick vs. thin)
Descriptive information, statistics, and annotation of top eyebrows (thick vs. thin) associated SNPs

Worksheet S6. Top SNPs associated with hair type (straight vs. curly)
Descriptive information, statistics, and annotation of top hair type (straight vs. curly) associated SNPs

Worksheet S7. Top SNPs associated with excessive hairiness
Descriptive information, statistics, and annotation of top excessive hairiness associated SNPs

Worksheet S8. Top SNPs associated with excessive sweating
Descriptive information, statistics, and annotation of top age excessive sweating associated SNPs

Supplementary Excel file #2 (Worksheets S9-S14): GWAS/eQTL colocalization analyses

First worksheet in file contains Legend with description of individual columns in the following worksheets:

Worksheet S9. Summary of ABF colocalization analysis of GWAS and multi-tissue eQTL data
Worksheet contains R coloc.abf function summary output for each colocalization analysis of a GWAS locus and multi-tissue eQTL data.

Worksheet S10. Per-SNP results of ABF colocalization analysis of GWAS and multi-tissue eQTL data
Per-SNP coloc.abf function results for GWAS locus/multi-tissue eQTL analyses shown in Worksheet S9 combined with GWAS and eQTL meta-information. Data was filtered to only include rows with 1) GWAS r^2>0.8, or 2) all moderate LD SNPs (r^2_{GWAS}>0.5 & r^2_{eQTL}>0.5) for loci/gene analyses with high confidence support for colocalization (PP.H4.abf>0.9).

Worksheet S11. Summary of ABF colocalization analysis of GWAS and single-tissue eQTL data
Worksheet contains R coloc.abf function summary output for each colocalization analysis of a GWAS locus and single-tissue eQTL data.

Worksheet S12. Per-SNP results of ABF colocalization analysis of GWAS and single-tissue eQTL data
Per-SNP coloc.abf function results for GWAS locus/single-tissue eQTL analyses shown in Worksheet S11 combined with GWAS and eQTL meta-information. Data was filtered to only include rows with 1) GWAS r^2>0.8, or 2) all moderate LD SNPs (r^2_{GWAS}>0.5 & r^2_{eQTL}>0.5) for loci/tissue-gene analyses with high confidence support for colocalization (PP.H4.abf>0.9).

Worksheet S13. SMR colocalization analysis of GWAS and multi-tissue eQTL data
Worksheet contains output from the SMR colocalization analysis method for a GWAS locus and multi-tissue eQTL data.

Worksheet S14. SMR colocalization analysis of GWAS and single-tissue eQTL data

Worksheet contains output from the SMR colocalization analysis method for a GWAS locus and single-tissue eQTL data.

Supplementary Figures

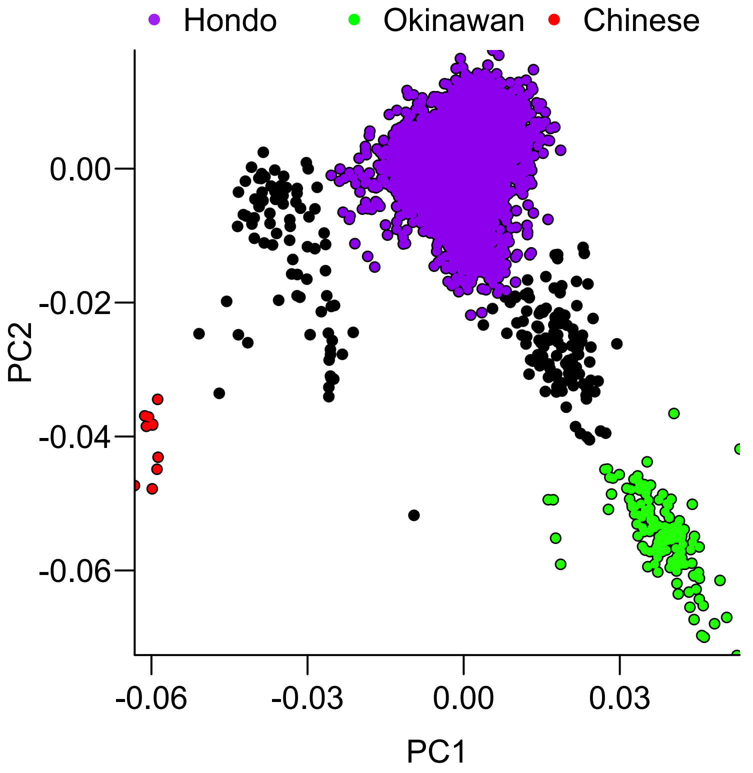

Figure S1. Population structure analysis.

PC1 and PC2 from PCA of East Asian samples after removal of outliers. Around the main Hondo population cluster, the extremes of local East Asian population structure are denoted by the Okinawan and Chinese (based on overlap with 1000G CHB samples) clusters.

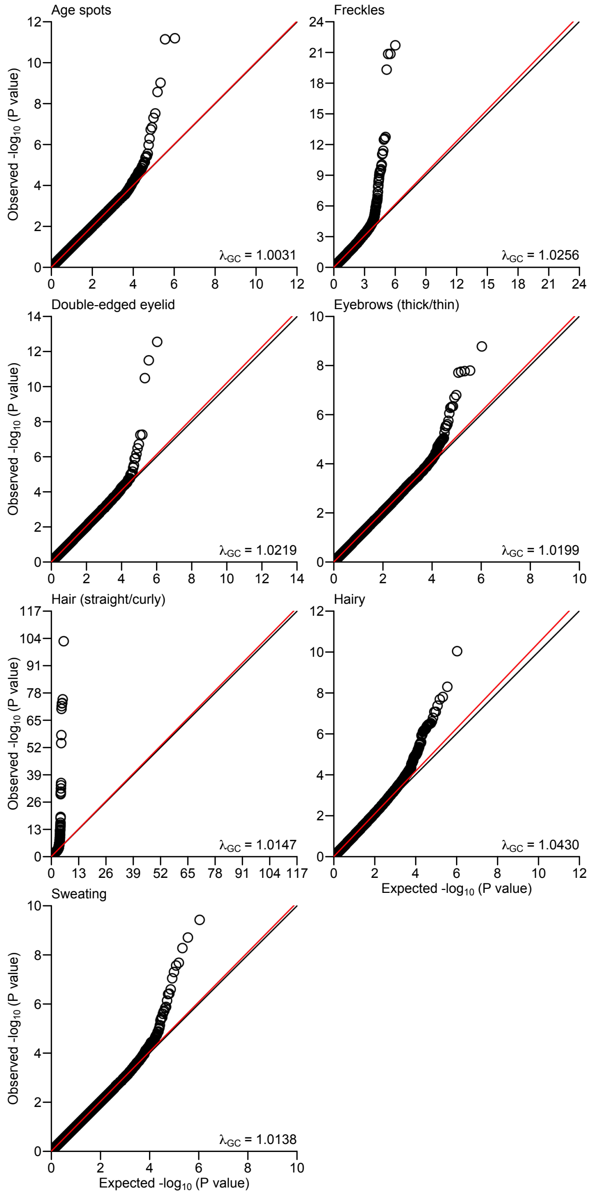

Figure S2. QQ plots of genome-wide meta-analysis association statistics.

QQ plot of expected and observed $-\log_{10}$ (P value) for genotyped GWAS statistics for seven skin phenotypes. We calculated λ_{GC} from the median observed χ^2 test statistics divided by the median of test statistics assuming a uniform distribution. Black line has slope of 1.0. Red line has slope of λ_{GC}.

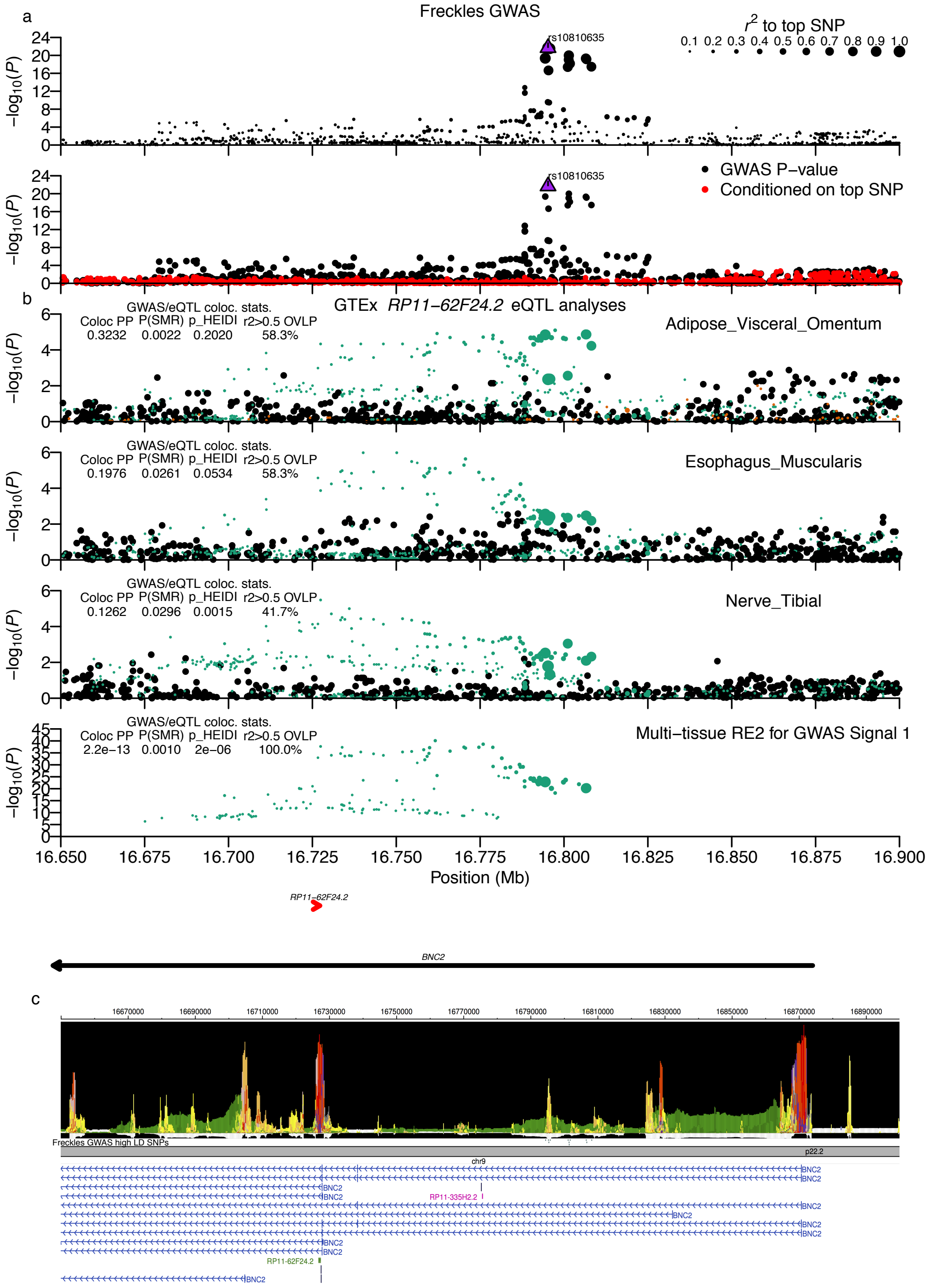

Figure S3. Chr9:16.79-16.81 Mb (*BNC2)* freckles locus.

Freckles associated variants in the chr9:16.79-16.81 Mb (*BNC2*) locus have weak/nominal evidence for colocalization with eQTLs for the ncRNA gene *RP11-62F24.2*. (**a**) Regional association plots of -log10(P-values), with the top sub-panel's points sized by r^2 to the top freckles SNP, and the bottom sub-panel showing SNPs with and without conditioning on the top SNP. GENCODE gene models are shown below association plots. The top GWAS SNP is plotted as an upright triangle. (**b**) shows -$\log_{10}$(P-values) for association with expression of *RP11-62F24.2* in GTExPortal single-tissue and multi-tissue Metasoft RE2 data. Points are sized by r^2 to the top GWAS SNP. Point colors denote different independent eQTL signals. Colocalization statistics from ABF and SMR methods and the percent of mod. LD (r^2>0.5) GWAS SNPs overlapping mod. LD eQTL SNPs are shown at the left of each sub-panel as an inlayed table. GENCODE gene models are shown below the eQTL plots. (**c**) presents output from the WashU EpiGenome Browser of an epilogos plot of the Roadmap Epigenomics 25-state imputed model of epigenetic states along with a track of high LD candidate causal variants and GENCODE transcript models in the region.

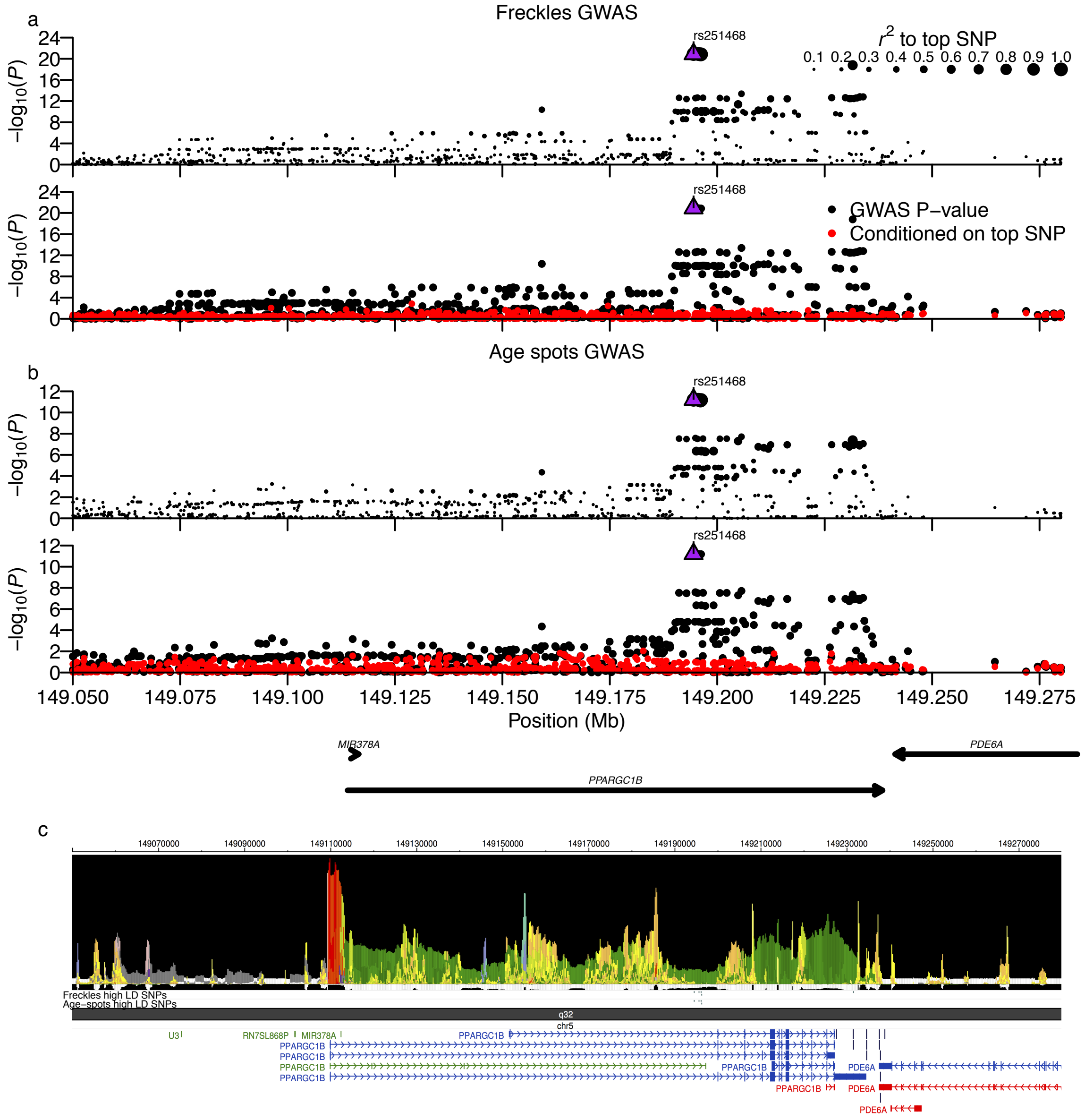

Figure S4. Chr5:149.19-149.23 Mb (*PPARGC1B*) skin-spots locus.

(**a-b**) Regional association plots of -log$_{10}$(P-values) in the Chr5:149.19-149.23 Mb (*PPARGC1B*) freckles/ age-spots associated locus. GWAS plot components are described in the Supplementary Figure S3 figure legend. (**c**) shows an epilogos plot of Roadmap Epigenomics 25-state imputed model of epigenetic states along with a track of high LD candidate causal variants for each skin-spot phenotype and GENCODE transcript models in the region.

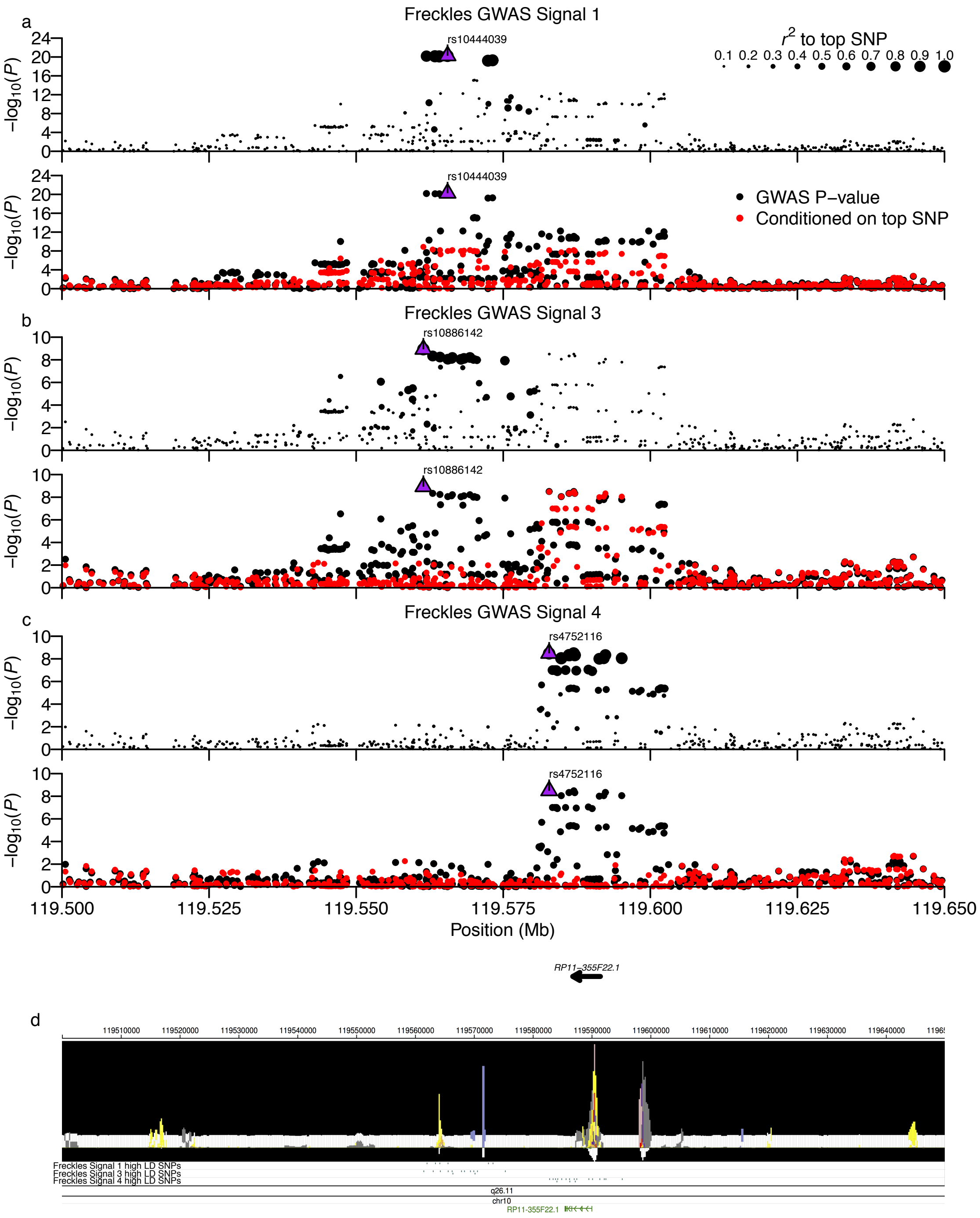

Figure S5. Chr10:119.52-119.63 Mb (*RAB11FIP2*) freckles multi-signal locus.

(**a**-**c**) Regional association plots of -$\log_{10}$(P-values) around three freckles association signals in the chr10:119.52-119.63 Mb (*RAB11FIP2*) locus. Regression conditioning was performed across a larger area that included another unlinked signal (Signal #2) that is shown in Figure 1. GWAS plot components are described in the Supplementary Figure S3 figure legend. (**d**) shows an epilogos plot of Roadmap Epigenomics 25-state imputed model of epigenetic states along with a track of high LD variants for each signal, and at the bottom, GENCODE transcript models in the region.

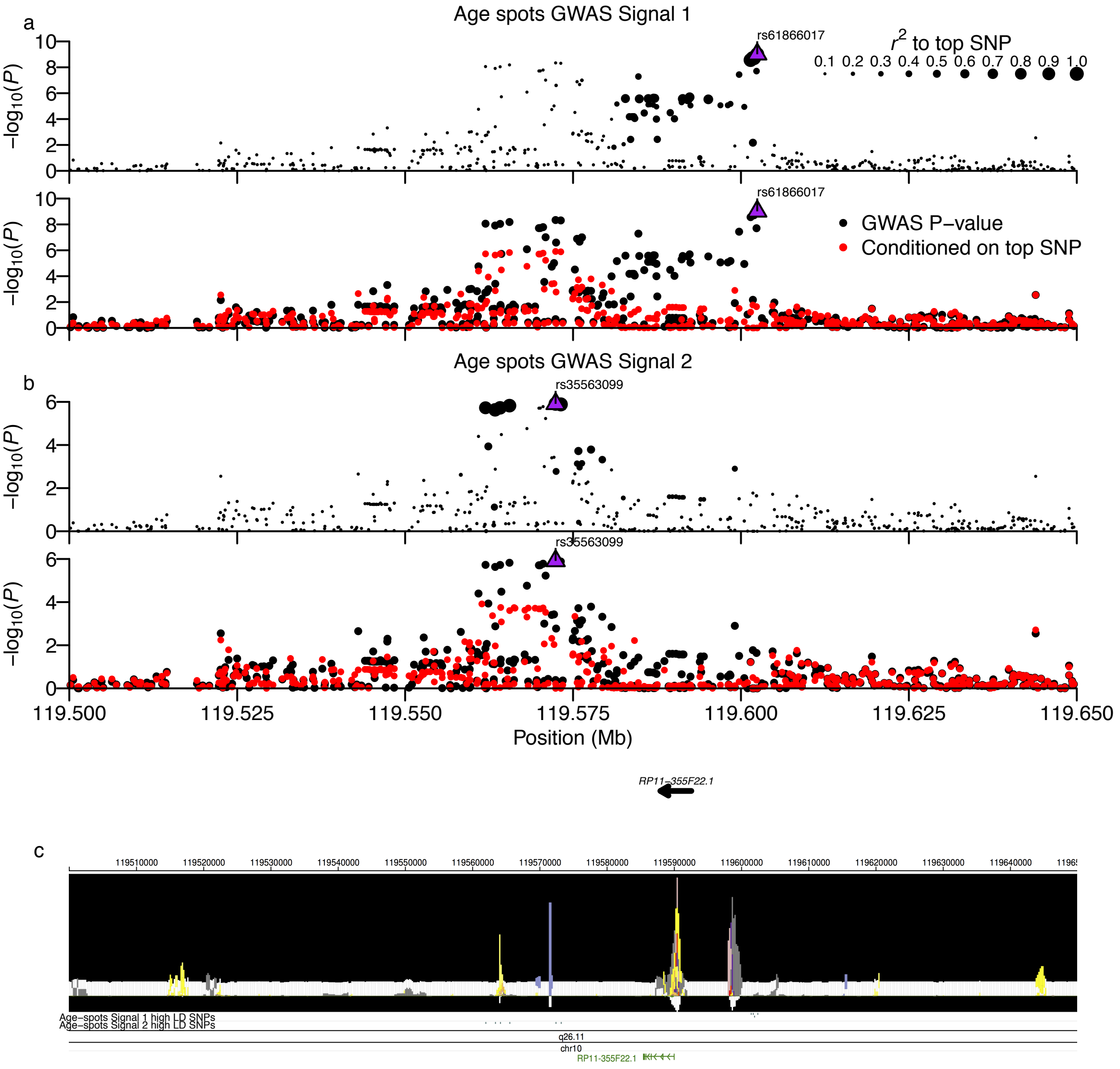

Figure S6. Chr10:119.52-119.63 Mb (*RAB11FIP2*) age spots multi-signal locus.

(**a**-**b**) Regional association plots of -$\log_{10}$(P-values) around two age spots association signals in the chr10:119.52-119.63 Mb (*RAB11FIP2*) locus. GWAS plot components are described in the Supplementary Figure S3 figure legend. (**c**) shows an epilogos plot of Roadmap Epigenomics 25-state imputed model of epigenetic states along with a track of high LD variants for each signal, and at the bottom, GENCODE transcript models in the region.

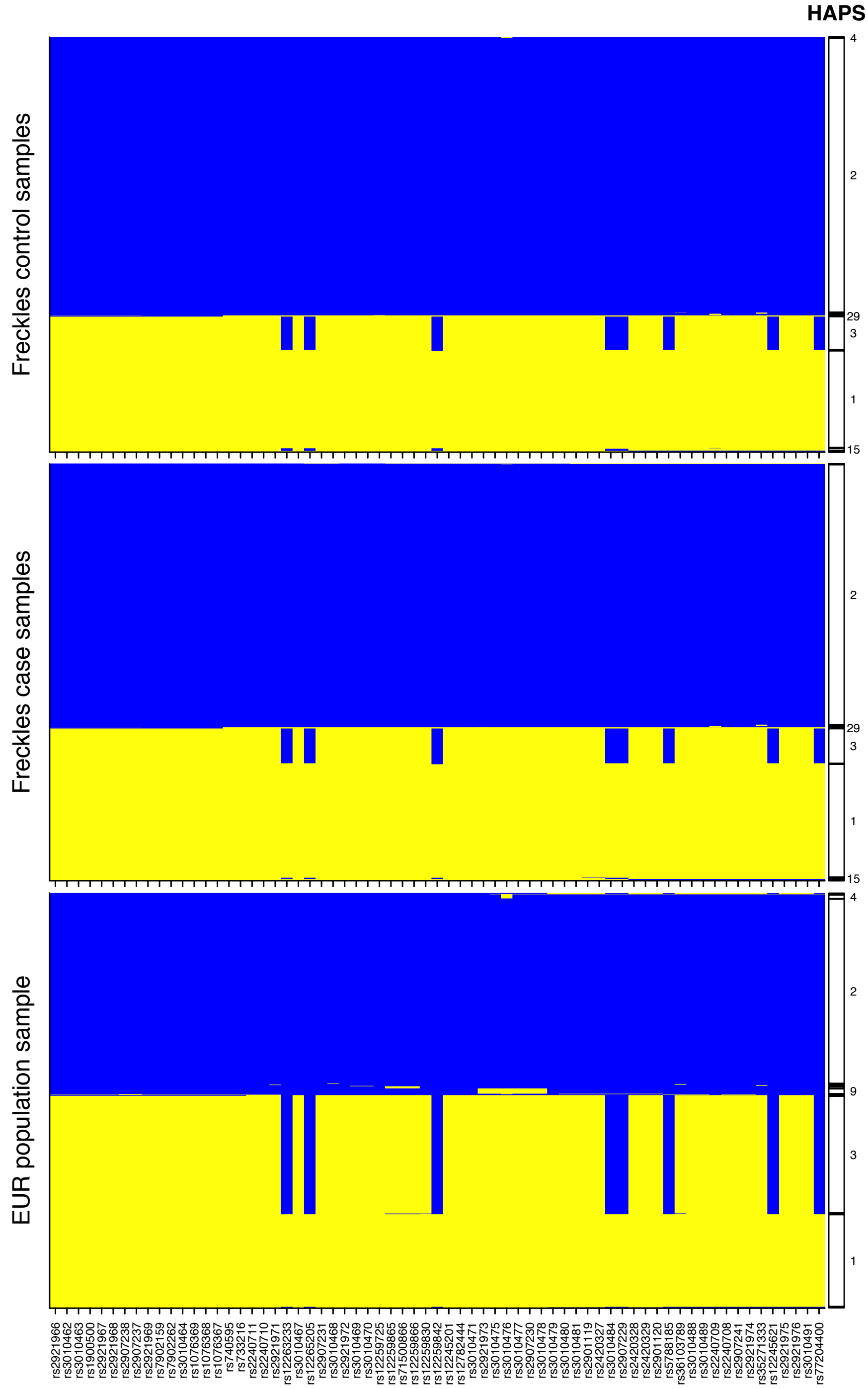

Figure S7. Chr10:118.45-118.48Mb (*HSPA12A*) freckles locus haplotype structure.

Haplotypes were determined by phasing GWAS and 1000G genotype data for SNPs that were in at least moderate LD by r^2 and high LD by *D'* (r^2>0.5 & P<1x10^{-8} & *D'*>0.98) to the top GWAS SNP. Haplotypes were then run through a hierarchical clustering algorithm to group haplotypes and then plotted as a matrix of cluster sorted haplotypes (y-axis) versus SNP rsids (x-axis). The minor allele in Japanese is plotted in yellow and the other allele in blue. The right hand y-axis labels haplotype cluster (HAPS).

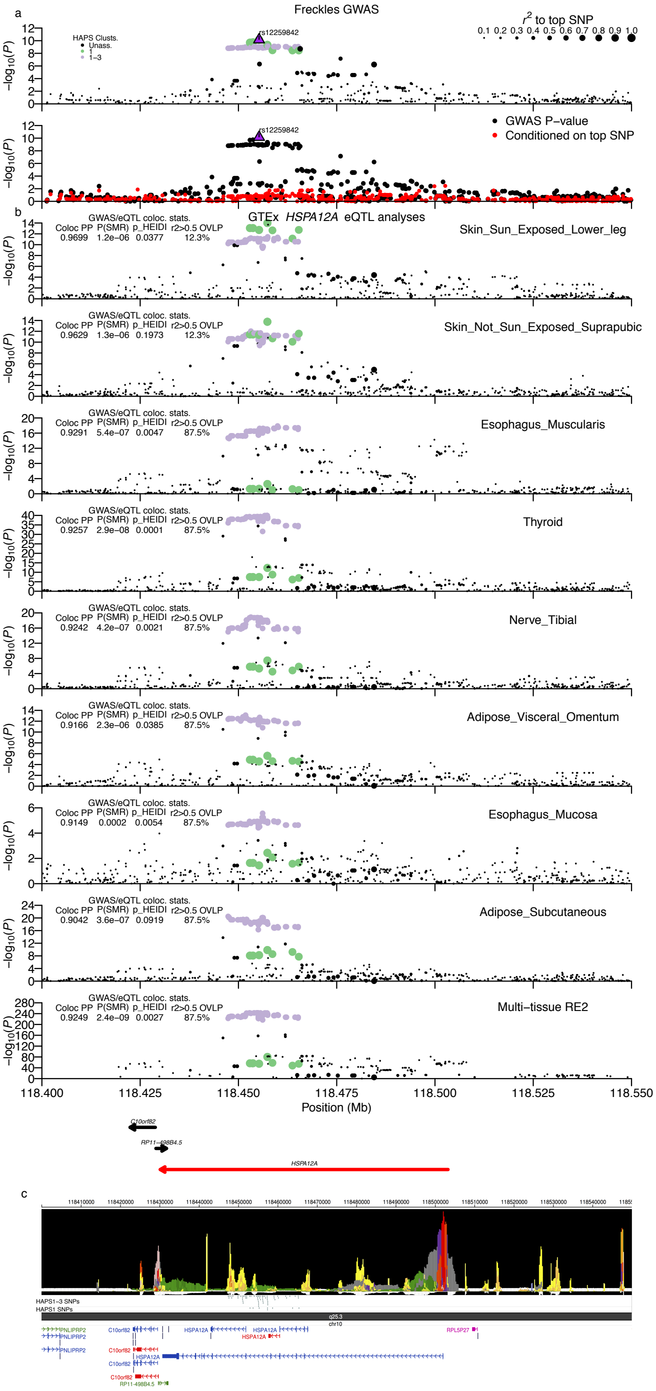

Figure S8. Chr10:118.45-118.48Mb (*HSPA12A*) freckles locus eQTL analysis.

Freckles association signal in the Chr10:118.45-118.48 Mb locus has strong evidence for colocalization with *HSPA12A* eQTLs. Of high LD SNPs (r^2>0.8 | r^2_{equiv}>0.8), a group with lower frequency is associated with *HSPA12A* expression only in the two GTEx skin tissue samples, while others with higher-frequency variants are associated in multiple tissues. (**a**) shows regional association plots of excessive sweating GWAS statistics are shown in (**a**). (**b**) shows colocalization analyses of the GWAS and GTExPortal single tissue or multi-tissue RE2 statistics. In the top-panel of (**a**) and each (**b**) sub-panel, SNPs are coloured if they were assigned to a haplotype cluster as shown in Figure S7. GWAS and eQTL plot components are otherwise as described in the Supplementary Figure S3 figure legend. (**c**) shows output from WashU EpiGenome Browser of an epilogos plot of the Roadmap Epigenomics 25-state imputed model of epigenetic states along with tracks of high LD variants divided by assigned haplotype cluster(s) and GENCODE transcript models in the region.

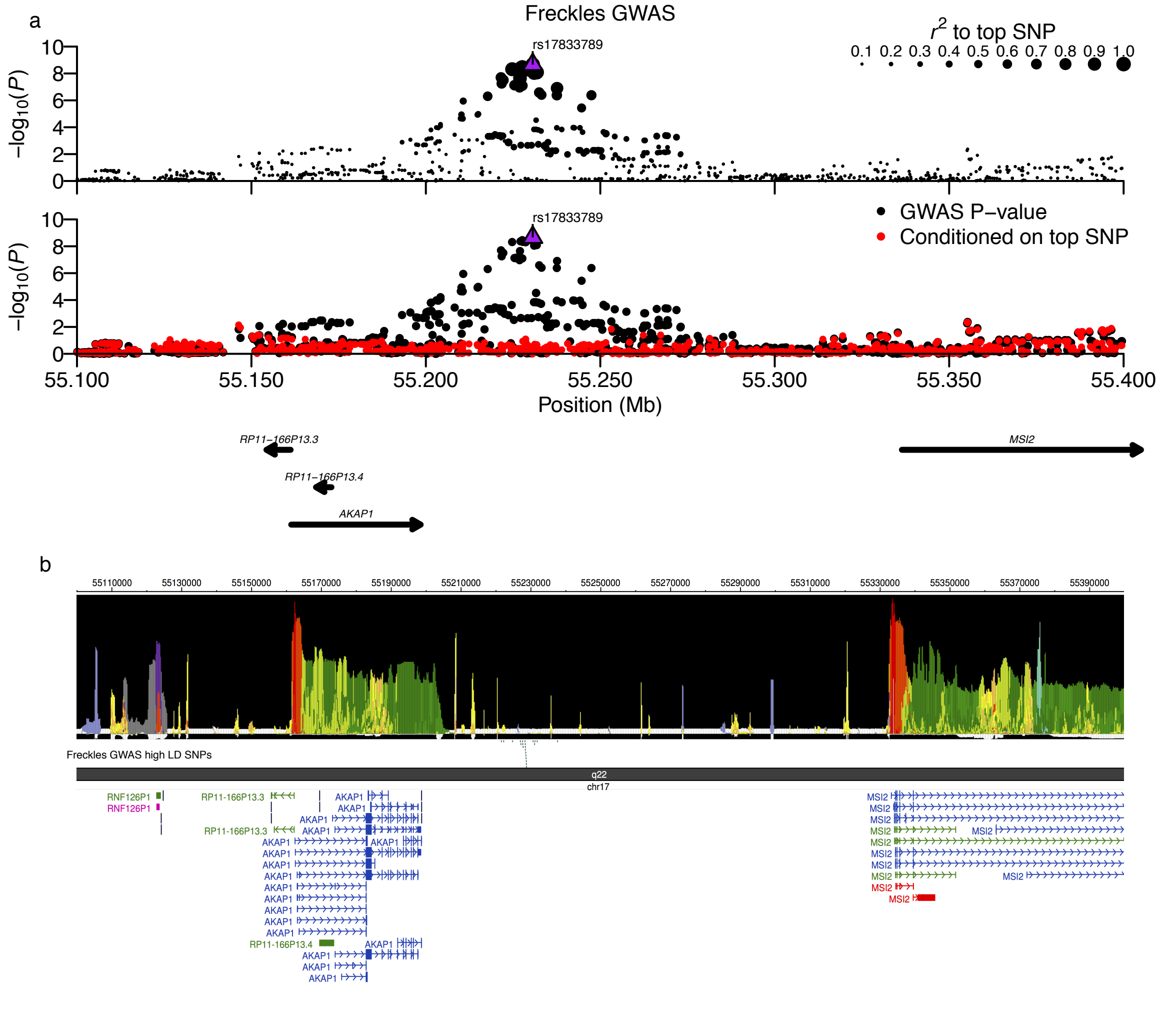

Figure S9. Chr17:55.21-55.25 Mb (*AKAP1*/*MSI2*) freckles locus.

(**a**) Regional association plots of -log$_{10}$(P-values) in the Chr17:55.21-55.25 Mb (*AKAP1*/*MSI2*) freckles association locus. GWAS plot components are described in the Supplementary Figure S3 figure legend. (**b**) shows WashU Epigenome Browser output with an epilogos plot of Roadmap Epigenomics 25-state imputed model of epigenetic states, a track of high LD freckles associated variants, and GENCODE transcript models in the region.

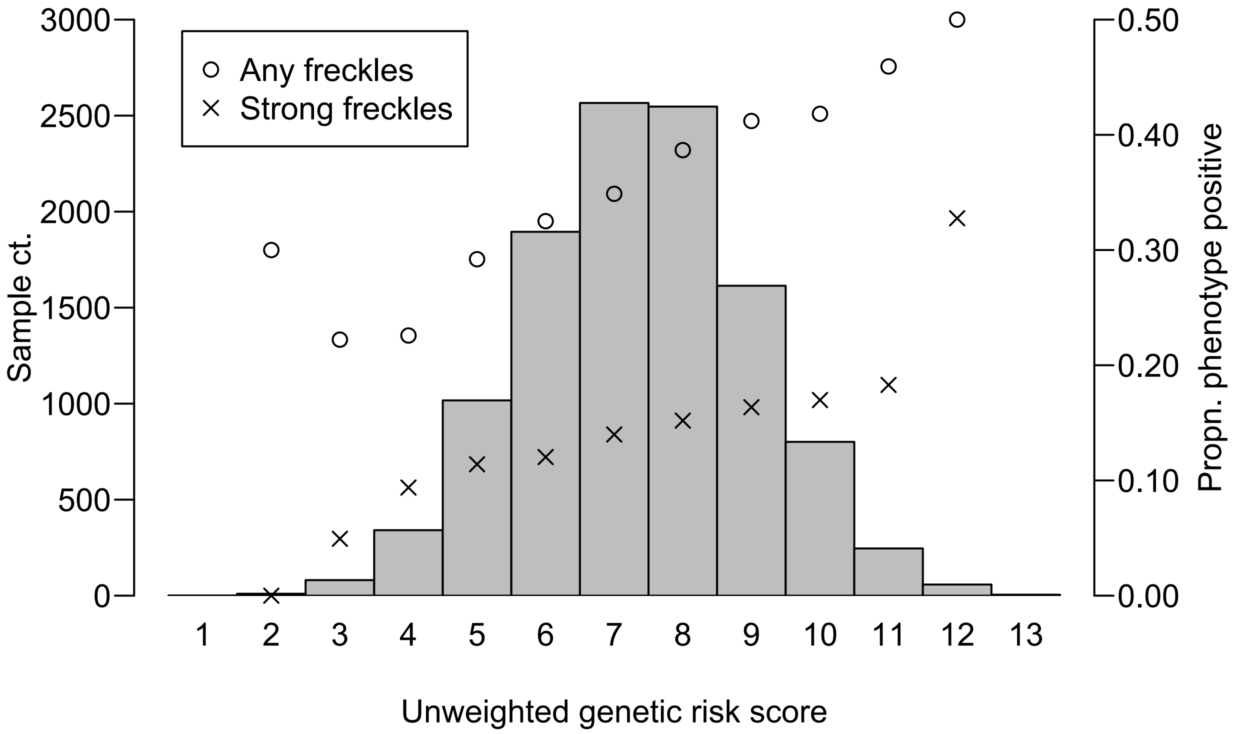

Figure S10. Genetic risk score (GRS) for freckles positivity.

An unweighted GRS was calculated from the glm models of freckles case-control status using the top SNP from each association signal identified in the freckles GWAS analysis. Plot shows the distribution of freckles GRS overlaid with the proportion of samples with any freckles (Freckles=Very applicable or Slightly applicable) or strong freckles (Freckles=Very applicable) in each GRS bin.

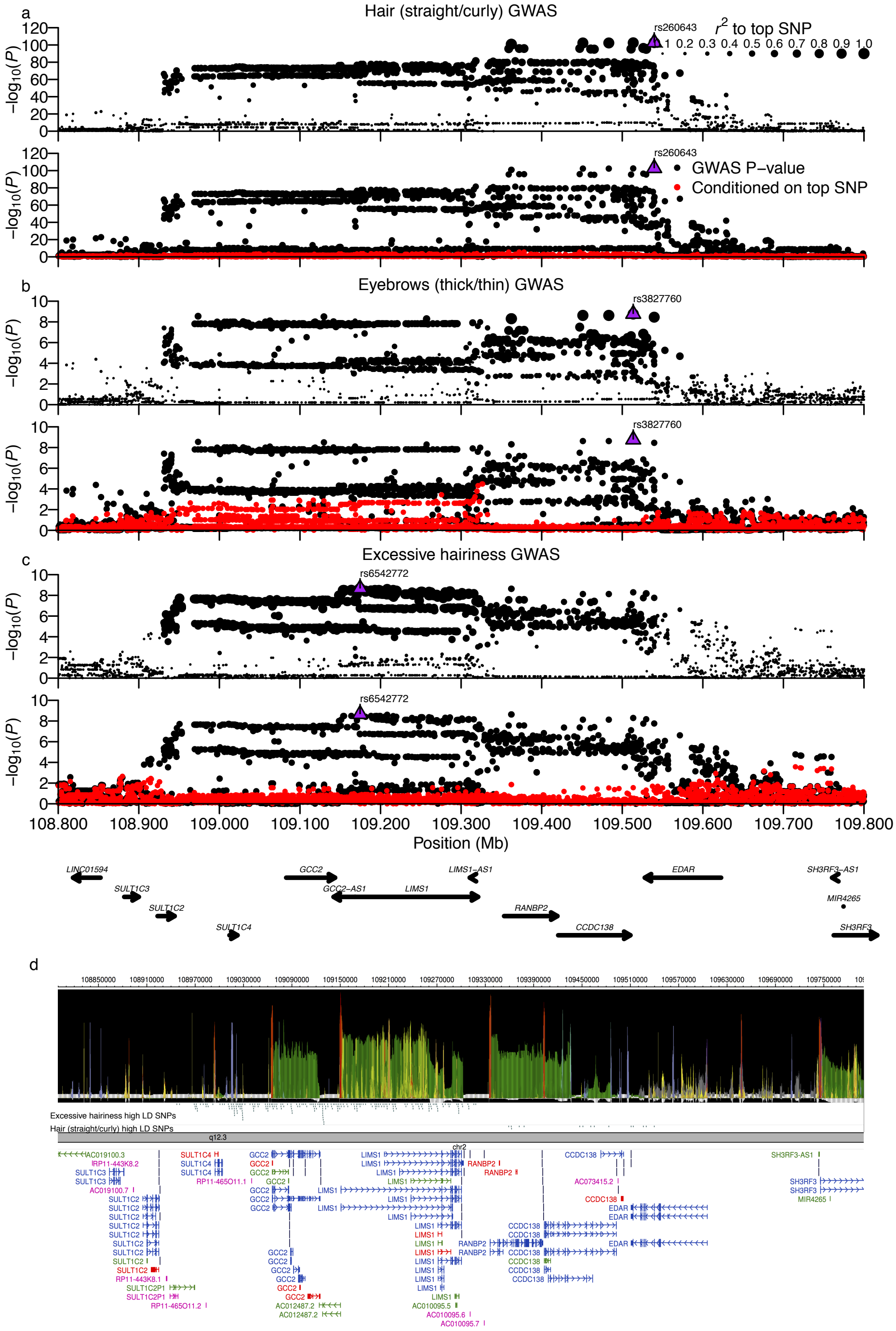

Figure S11. Chr2:108.93-109.57 Mb (*EDAR*/*GCC2*/*LIMS1*) hair morphology and density loci.

(**a**-**c**) Regional association plots of -log$_{10}$(P-values) around hair morphology and density association signals in the chr2:108.93-109.57 (*EDAR*/*GCC2*/*LIMS1*) locus. (**a**) Hair (straight/curly). (**b**) Eyebrow thickness. (**c**) Excessive hairiness. GWAS plot components are described in the Supplementary Figure S3 figure legend. (**d**) shows an epilogos plot of Roadmap Epigenomics 25-state imputed model of epigenetic states along with a track of high LD variants for each signal, and at the bottom, GENCODE transcript models in the region.

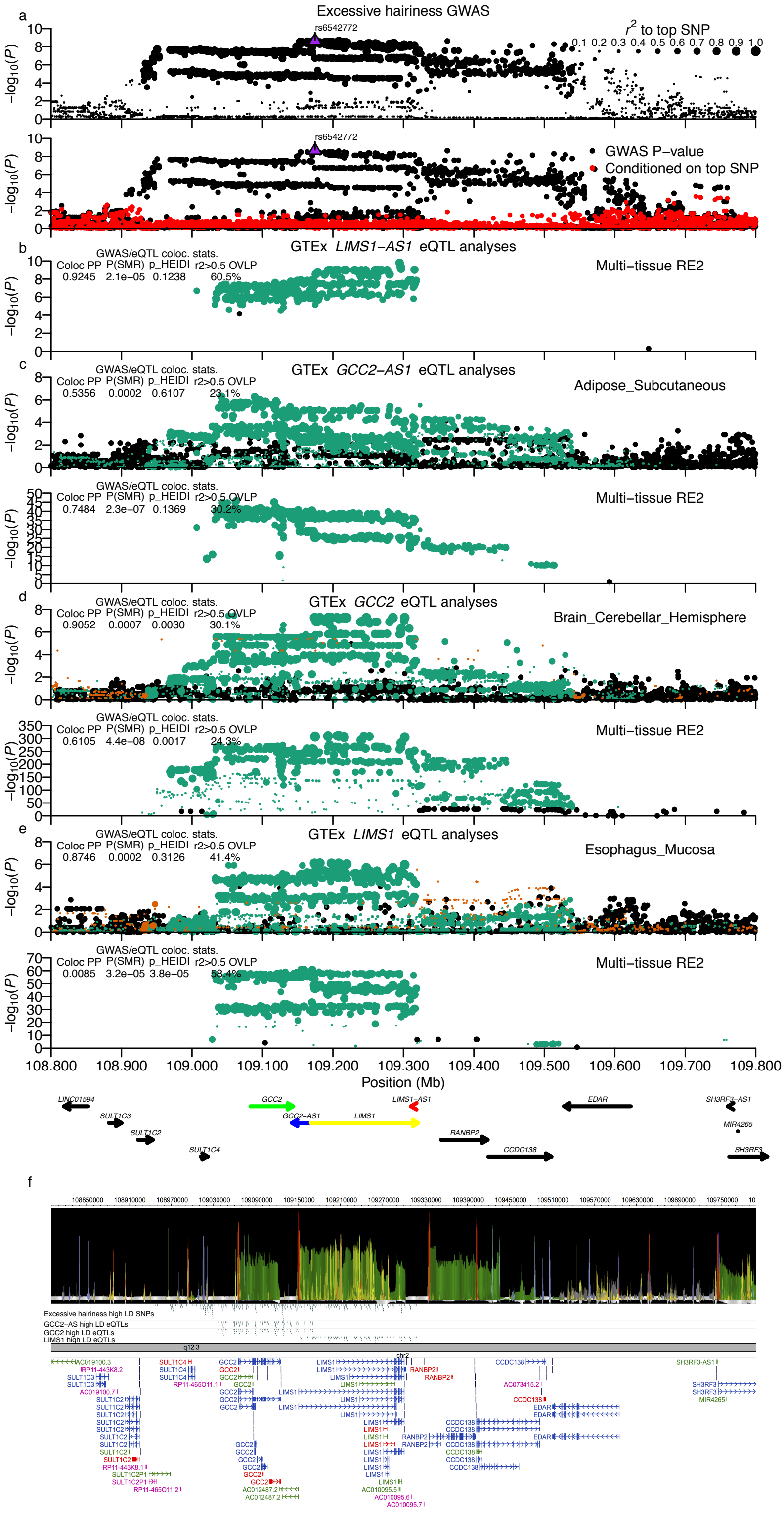

Figure S12. Chr2:108.93-109.57 Mb (*GCC2*/*LIMS1*) excessive hairiness locus eQTL analysis.

Excessive hairiness associated SNPs in the Chr2:108.93-109.57 Mb locus possess moderate to strong evidence for colocalization with eQTLs for *LIMS1* and *GCC2* and the antisense ncRNA genes *LIMS-AS1* and *GCC2-AS1*. Regional association plots of excessive sweating GWAS statistics are shown in (**a**). (**b-e**) show colocalization analyses of the GWAS and GTExPortal single tissue or multi-tissue RE2 statistics. GWAS and eQTL plot components are described in the Supplementary Figure S3 figure legend. (**f**) shows output from WashU EpiGenome Browser of an epilogos plot of the Roadmap Epigenomics 25-state imputed model of epigenetic states along with tracks of high LD GWAS or high LD eQTL variants and GENCODE transcript models in the region.

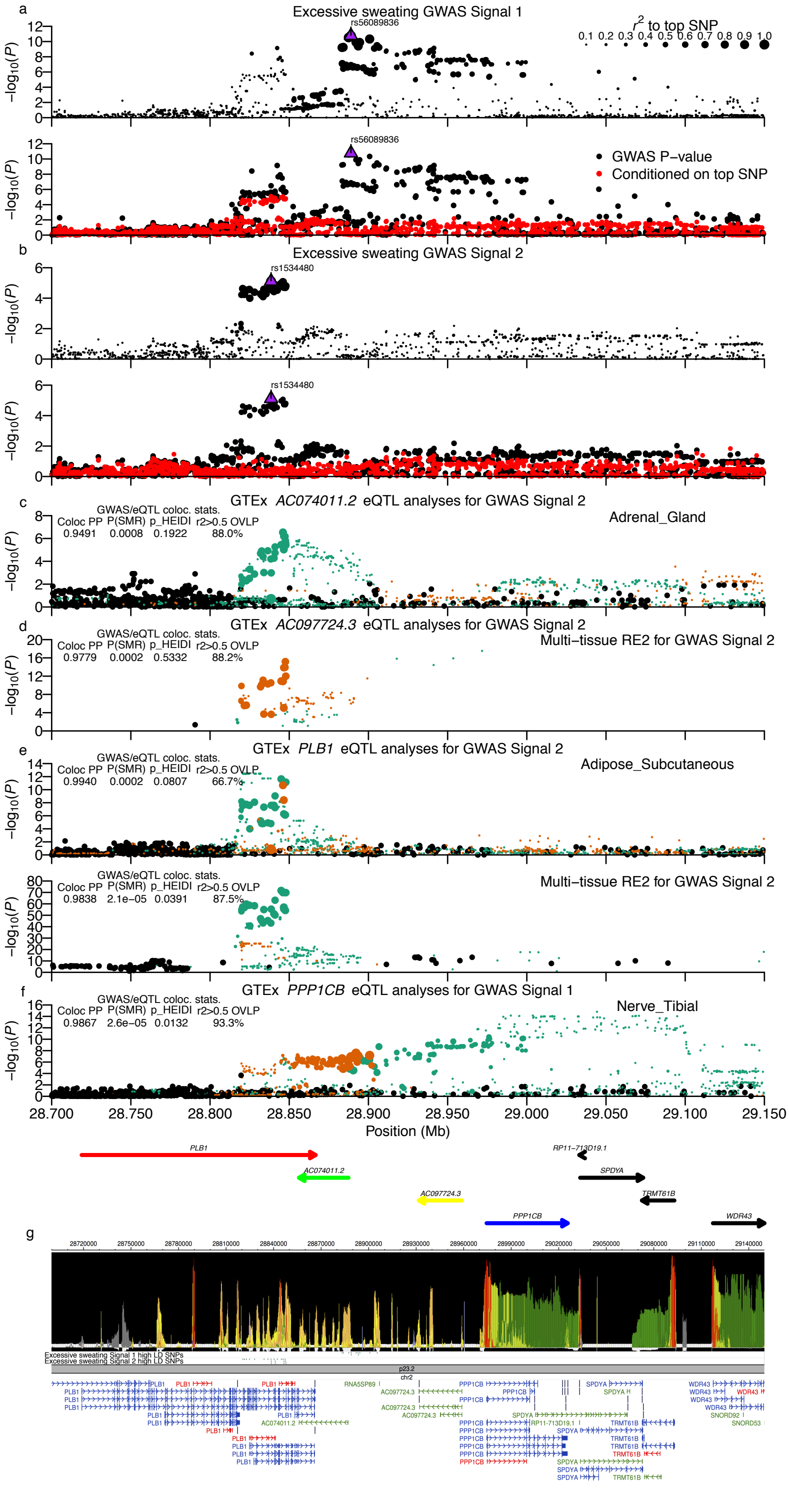

Figure S13. Chr2:28.82-29.05 Mb (*PLB1/PPP1CB*) excessive sweating locus eQTL analysis.

Signal #2 in the Chr2:28.82-29.05 Mb excessive sweating locus has strong evidence for colocalization with eQTLs for *PLB1* and the ncRNA genes *AC074011.2* and *AC097724.3*, while Signal #1 has strong support for overlap with *PPP1CB* eQTLs in a single tissue (Tibial nerve). Regional association plots of excessive sweating GWAS statistics are shown in (**a**) for Signal #1 and in (**b**) for Signal #2. (**c**-**f**) show colocalization analyses of the GWAS and either GTExPortal single tissue or multi-tissue RE2 statistics in *AC074011.2*, *AC097724.3*, *PLB1* , and *PPP1CB*. GWAS and eQTL plot components are described in the Supplementary Figure S3 figure legend. (**g**) shows output from WashU EpiGenome Browser of an epilogos plot of the Roadmap Epigenomics 25-state imputed model of epigenetic states along with tracks of high LD signal 1 and signal 2 variants and GENCODE transcript models in the region.

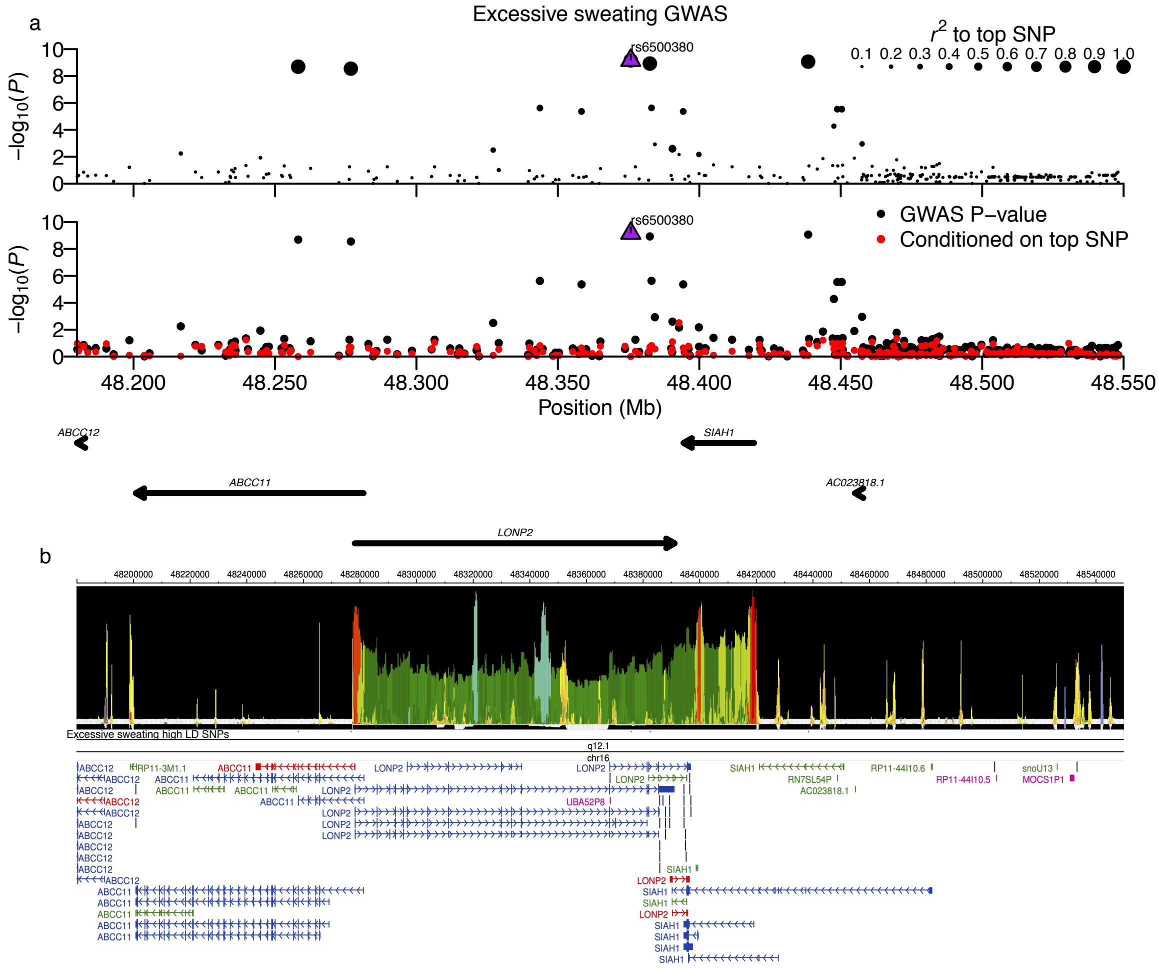

Figure S14. Chr16:48.26-48.45 Mb (*ABCC11*/*LONP2*) excessive sweating locus.

(**a**) regional association plots of -$\log_{10}$(P-values) around the Chr16:48.26-48.45 Mb (*ABCC11*/*LONP2*) excessive sweating locus. GWAS plot components are described in the Supplementary Figure S3 figure legend. (**b**) presents output from the WashU EpiGenome Browser of an epilogos plot of the Roadmap Epigenomics 25-state imputed model of epigenetic states along with a track of high LD candidate causal variants and GENCODE transcript models in the region.
